# Identification of Two Common Bottlenose Dolphin (*Tursiops truncatus*) Ecotypes in the Guadeloupe Archipelago, Eastern Caribbean

**DOI:** 10.3390/ani15010108

**Published:** 2025-01-05

**Authors:** Rachel Haderlé, Laurent Bouveret, Bruno Serranito, Paula Méndez-Fernandez, Olivier Adam, Mélodie Penel, Jérôme Couvat, Iwan Le Berre, Jean-Luc Jung

**Affiliations:** 1 Institut de Systématique, Évolution, Biodiversité (ISYEB), Muséum National d’Histoire Naturelle, CNRS, Sorbonne Université, EPHE-PSL, Université des Antilles, 75005 Paris, France; 2 Station Marine de Dinard du Muséum National d’Histoire Naturelle, 35800 Dinard, France; 3 Observatoire des Mammifères Marins de l’Archipel Guadeloupéen (OMMAG), 97117 Port-Louis, France; 4 Laboratoire de Biologie des Organismes et des Ecosystèmes Aquatiques (BOREA UMR 7208), MNHN, CNRS 8067, Sorbonne Université, IRD 207, UCN, Université des Antilles, 75005 Paris, France; 5 Observatoire Pelagis, UAR 3462 La Rochelle Université—CNRS, 17000 La Rochelle, France; 6 Institut d’Alembert UMR 7190, LAM, Sorbonne University, CNRS, 75005 Paris, France; 7 Campus de Fouillole, Université des Antilles et de la Guyane, 97159 Pointe-à-Pitre, France; 8 Sanctuaire Agoa pour les Mammifères Marins, Office Français de la Biodiversité, 97229 Les Trois Ilets, France; 9 UMR LETG-Brest CNRS-6554, IUEM Université de Bretagne Occidentale, 29280 Brest, France

**Keywords:** *Tursiops truncatus*, ecotypes, Caribbean, coastal, oceanic, photo-identification, mitochondrial DNA, habitat modeling, morphotype, maritime traffic

## Abstract

The common bottlenose dolphin (*Tursiops truncatus*) exhibits significant intraspecific diversity globally, with distinct ecotypes identified in various regions. In the Guadeloupe archipelago, the citizen science NGO OMMAG has been monitoring these dolphins for over a decade, documenting two distinct morphotypes. This study investigates whether these morphotypes represent coastal and oceanic ecotypes, which have not been previously identified in the region. We characterized morphological differences between the two morphotypes, analyzed genetic variation in individuals stranded around Guadeloupe over the past ten years, and modeled their habitats. Results revealed that these morphotypes align with the ecotypes described in the Atlantic Ocean, forming two distinct genetic groups corresponding to Caribbean ecotypes. Habitat modeling showed differences in habitat preferences between the morphotypes. These findings provide strong evidence for the existence of two distinct ecotypes of *T. truncatus* in Guadeloupe. Considering the varied risks of exposure to maritime traffic, our study suggests that these ecotypes should be managed as separate units within the species to better inform conservation strategies in the region.

## Simple Summary

This study examines the intraspecific diversity of common bottlenose dolphins (*Tursiops truncatus*) around Guadeloupe. Using over a decade of photo-identification data, two morphotypes of common bottlenose dolphins were identified in the Guadeloupe archipelago. This study conducted a detailed analysis of their morphological differences, genetic diversity, and habitat preferences. DNA samples from stranded individuals were compared with ecotype-specific sequences from other regions to assess local genetic differentiation, while habitat modeling was applied to identify spatial patterns associated with each morphotype. Based on differences in morphology, genetics, and habitat preferences, the findings demonstrate the presence of two ecotypes in Guadeloupe, corresponding to the coastal and oceanic *T. truncatus* ecotypes. As a case study, the analysis of exposure to maritime traffic suggests differential risk levels between the two ecotypes. This research underscores the importance of considering ecotype-specific characteristics in marine mammal conservation planning, particularly in the context of a marine protected area dedicated to marine mammal conservation, such as the Agoa Sanctuary.

## 1. Introduction

Among cetaceans, several groups of the same species may adapt and evolve independently due to behavioral specializations that limit gene flow (e.g., [1,2,3,4]). As a result, patterns in genetic population structure are often shaped by behavioral, social, or cultural traits [3,5,6,7,8]. Genetic diversity, correlated with intraspecific variations in spatial distribution, foraging ecology, and social structure, may lead to the emergence of ecotypes. First introduced in plants [9], the concept of “ecotype” is complex, partly due to its interdisciplinary nature, but it has proven highly valuable for evolutionary biology and applied conservation [10]. This complexity has led to some confusion and inconsistencies in the literature, especially regarding the criteria used to define ecotype status [11,12]. A commonly accepted definition is that of Lowry [11], which is also used by Louis et al. [3], which considers ecotypes as groups of populations that differ in genetic traits as well as ecological and/or physiological characteristics. Building on this, Stronen et al. [10] outlined a framework to distinguish ecotypes, based on three conditions: phenotypic variations, differences in preferred habitats, and genetic distinction, which must be observed for groups of the same species to qualify as ecotypes.

Today, several marine mammal species are known to include ecotypes. Several ecotypes of killer whales (*Orcinus orca*) have been described, often associated with feeding specialization. In the Northeast Pacific, resident, transient, and offshore ecotypes are distinguished by multiple independent lines of evidence [13] based on genetic, morphological, geographical, and dietary characteristics [12]. The differences between ecotypes are so pronounced that Morin et al. [14] revised the taxonomy of two eastern North Pacific ecotypes, which are now classified as distinct subspecies: *O. orca ater* (resident killer whale) and *O. orca rectipinnus* (Bigg’s killer whale, also known as the transient ecotype). The Antillean manatee (*Trichechus manatus manatus*) also exhibits distinct ecotypes, influenced by its diverse habitats. Comparisons of body condition indices revealed differences between manatees in freshwater ecosystems and those in coastal and marine areas. These variations suggest that the subspecies comprises at least two ecotypes—riverine and coastal—each facing different fitness tradeoffs due to environmental and resource limitations [15].

The common bottlenose dolphin (*Tursiops truncatus*) is another example of a marine mammal species exhibiting highly complex intraspecific diversity. *T. truncatus* is a cosmopolitan species found in tropical to temperate waters, both in coastal and estuarine ecosystems as well as in the open ocean. Currently, four subspecies of *T. truncatus* are officially recognized [16]. In addition, two ecotypes of *T. truncatus* have been described over the past several decades [3,17,18,19,20,21,22,23,24,25]. Often referred to as coastal and oceanic, the ecotypes of *T. truncatus* have been distinguished based on various independent lines of evidence, which do not always meet all three criteria defined by Stronen et al. [10]: hematological profiles [26], parasite load [27], skull morphology [1,28], genetic structure [4,29,30,31], and morphology (see Table S1). In the Atlantic, in terms of morphology, the oceanic ecotype of *T. truncatus* is generally larger (total length, skull length, and internal nostril width), darker, has a more falcate dorsal fin, and has a shorter rostrum than the coastal ecotype [29,32,33,34]. *T. truncatus* ecotypes seem to be almost ubiquitous, having been widely detected from the Atlantic to the Pacific. However, there are counterexamples. For instance, *T. truncatus* ecotypes were not detected in the Azores by Quérouil et al. [35]. In the northern Caribbean Sea, Caballero et al. [36] studied the genetic diversity of *T. truncatus* in a region encompassing the Gulf of Mexico, Honduras, Colombia, the US Virgin Islands, Puerto Rico, Jamaica, Cuba, and the Bahamas. Mitochondrial DNA polymorphisms demonstrated the regional presence of at least two genetically differentiated forms of *T. truncatus*, the coastal ecotype and the worldwide distributed form, which may correspond to the oceanic ecotype. These results have been confirmed in other regions of the Caribbean, such as La Guajira (Colombia), Panamá (Bocas del Toro), and Costa Rica [37,38].

Whether the *T. truncatus* ecotypes are on distinct evolutionary trajectories remains an open question [1]. In fact, ecotypes may coincide with distinct taxonomic units, such as subspecies or species [39]. To formulate a taxonomic hypothesis in cetaceans, such as delimiting subspecies, authors must specify the indices used as the basis for the hypothesis and those employed to stratify the data [13]. These indices often include discrepancies in geographic distribution, morphology, ecology, or acoustics, facilitating the review of several pertinent independent lines of evidence [13,17]. The framework proposed by Stronen et al. [10] aligns with this approach for ecotype delimitations. The *T. erebennus* species, the most recently recognized species of the genus, was first identified as the coastal ecotype of *T. truncatus* in the Northwest Atlantic [1]. Coastal and oceanic ecotypes of *T. truncatus* exhibit marked genetic differentiation, which can sometimes be greater than that observed between (sub)species (e.g., [18]).

Distinguishing ecotypes in *T. truncatus* is therefore of fundamental interest for understanding the genetic diversity of this highly mobile cetacean species with an extensive home range. It is equally critical for the effective management and conservation of the species, especially where local concerns are known.

Given its cosmopolitan distribution, *T. truncatus* is globally classified as Least Concern (LC) by the IUCN. However, several local populations are threatened, as demonstrated, for instance, in New Zealand [40]. Additionally, other groups, which appear to be highly isolated, of low abundance, and with restricted home ranges, require intensive monitoring. Notable examples include populations around the island of Sein in Brittany [41] and in the Bay of Setúbal in Portugal [42]. While this species has been extensively studied in some regions, targeted research is therefore strongly needed in other areas to address knowledge gaps and inform effective conservation strategies.

The Guadeloupe Archipelago, located at the heart of the Agoa Sanctuary—a vast marine protected area covering the entire French exclusive economic zone of the Lesser Antilles and dedicated to the protection and conservation of marine mammals—serves as a typical example of a place where comprehensive studies of *T. truncatus* ecotypes are essential. A major part of the present knowledge about the species comes from the surveys conducted since 2011 by the “*Observatoire des Mammifères Marins de l’Archipel Guadeloupéen*” (OMMAG), an NGO dedicated to citizen science projects [43]. Over a decade of photo-identification monitoring has revealed the presence of two distinct morphotypes (groups of individuals from the same species sharing specific morphological characteristics) of *T. truncatus* in the archipelago. However, whether these morphotypes correspond to distinct coastal and oceanic ecotypes, as described in other regions, remains uncertain.

To address this question, this study aims to determine whether the two observed morphotypes of *T. truncatus* correspond to distinct coastal and oceanic ecotypes. To achieve this, we (i) characterized the morphological differences between the two morphotypes, (ii) looked for the existence of genetic variation in *T. truncatus* individuals stranded around Guadeloupe in the last ten years and correlated these variations with ecotype-specific DNA data published by others in Genbank, and (iii) modeled the habitat of both morphotypes around Guadeloupe in order to detect potential differences in preferential habitats. Additionally, based on previous studies analyzing the maritime traffic around Guadeloupe [44,45], we highlighted differential exposure risks for the two ecotypes of *T. truncatus* around Guadeloupe.

This study is the first to investigate the presence of coastal and oceanic *T. truncatus* ecotypes within the Agoa Sanctuary, providing novel insights into their morphology, genetics, and habitat preferences. By identifying ecotype-specific risks, we emphasize the need to integrate these findings into conservation strategies tailored to the Guadeloupe Archipelago. To implement these objectives, we employed an integrated approach combining photographic, genetic, and ecological data analysis.

## 2. Materials and Methods

To achieve our objectives, we employed an integrated approach combining photographic, genetic, and ecological data analyses. Specifically, this section will first address the photographic data used for morphological analysis, followed by the genetic data to investigate population structure, and finally, habitat modeling to assess morphotype preferences.

### 2.1. Photographic Data

Photographs and videos of *T. truncatus* were taken by the OMMAG members from small boats using Nikon D300S, D500, and D7200 digital cameras (Nikon Corporation, Tokyo, Japan) equipped with 70–200 mm lenses. These photographs were classified and sorted according to a set of parameters (proportion of visible dorsal fin, focus, angle of view, exposure, and image magnification) to retain only those of sufficient quality for photo-identification.

### 2.2. Genetic Analysis

Throughout the year, data and samples are collected from stranded dead cetaceans around Guadeloupe by the NGO Evasion Tropicale, the Guadeloupean correspondent of the French National Stranding network coordinated by the Pelagis observatory (UAR 3462). For *T. truncatus*, muscle and other internal tissue samples have been taken from 10 stranded individuals between 2013 and 2023. This limited number is due to the low number of strandings occurring around the Guadeloupean archipelago. In addition, an opportunistic sample collected by the Saint Martin Stranding Network (coordinated by the Réserve Naturelle de Saint Martin) on a common bottlenose dolphin stranded near Saint Martin Island in 2019 was added to this study. No morphological comparison of individuals from which samples were collected was performed because the ecotypes were not known, and no comparative measurements were linked.

#### 2.2.1. Sequencing of the Mitochondrial Control Region (MCR)

Genomic DNA was extracted from all the samples using the DNeasy Blood and Tissue Kit (Qiagen, Hilden, Germany) following the manufacturer’s recommendations. The quality of the extracted DNA was checked by agarose gel electrophoresis. Primers Dlp1.5 (5′-TCACCCAAAGCTGRARTTCTA-3′) and Dlp8G (5′-GGAGTACTATGTCCTGTAACCA-3′) [46] were used to amplify a 674 bp fragment of the mitochondrial control region (MCR) using the conditions described in Girardet et al. [47]. PCR products were sequenced by a provider (Eurofins Genomics, Ebersberg, Germany) on both strands in the presence of one of the PCR primers. Sequences were edited and aligned with Geneious Prime 2019.2.3 [48] to produce consensus sequences containing no uncertainties. Individual consensus sequences were aligned, producing the “dataset 1” (676 bp).

#### 2.2.2. Construction of Extended Datasets

Sequences were compared to those available in GenBank using BLAST [49] to search for similar data. An extended dataset, named “Caribbean” (see Table S2), was then constructed, composed of 44 haplotypes of *T. truncatus* (24 from the coastal ecotype and 20 from the oceanic one), all determined from samples taken in the Caribbean, the Gulf of Mexico, Honduras, Colombia, Panama, and Costa Rica [36,37]. All these sequences share a common 288 bp fragment. Another dataset, further extended, named “Atlantic” (see Table S3), was constructed by adding to the “Caribbean” dataset 71 haplotypes from the Northwest Atlantic (NWA [50]), 23 from the Southwest Atlantic (SWA [32]), 67 from the Northeast Atlantic (NEA [3]), and 126 from the Azores and Madeira [35]. All these sequences share the same 289 bp common fragment. All the haplotypes contained in the Caribbean and the Atlantic datasets were assigned to the coastal or the oceanic ecotype based on the information available in the publications, with the only exception of data from Quérouil et al. [35], as ecotypes were not identified in the Azores.

#### 2.2.3. MCR Sequences Data Analysis, Haplotype Network, and Phylogenetic Tree Construction

For each dataset, the number of haplotypes (H), number of polymorphic sites (S), haplotype diversity (*Hd*), and nucleotide diversity (*π*) were determined using ARLEQUIN 3.5 [51]. Fu’s *Fs* [52] and Tajima’s *D* [53] were estimated using DnaSP6 version 6.12.03 software [54]. To estimate genetic differentiation among groups, *Fst* and *Φst* indexes were computed using ARLEQUIN 3.5 [51], and the nearest neighbour statistic (*Snn*) index was based on a sequence distance matrix [55] using DnaSP6 [54]. Random permutations were used to assess the statistical significance of *Fst* and *Φst* (*n* = 100) and *Snn* (*n* = 1000).

To construct phylogenetic trees, the most suitable substitution model was determined using MEGA11 [56]. The model was selected based on the lowest Bayesian Information Criterion (BIC) and corrected Akaike Information Criterion (AICc) values. An outgroup was incorporated into the analysis (*Stenella attenuata* accession number KX857264). The phylogenetic tree was reconstructed using the IQ-TREE web service platform [57] with bootstrap support set to 1000 generations, ensuring robust branch reliability. The resulting trees were visualized using the iTOL (Interactive Tree of Life) tool [58]. All branches were maintained during the tree construction process.

Median-joining networks were constructed with the number of mutations as distances using Network 10.2 software (https://fluxus-engineering.com).

### 2.3. Method for Analyzing Preferential Habitats

The preferential habitat of both *T. truncatus* morphotypes was analyzed using all the georeferenced photographs taken between 2012 and 2022 around Guadeloupe. Points of observation for both morphotypes were plotted, and the overlap between their distributions was computed using Schoener’s *D* statistic [59] with the ecospat package [60]. The distribution patterns were also compared using two permutation tests [61]: a similarity test and an equivalence test.

The distribution of each morphotype was further studied using species distribution models. A set of selected environmental variables selected on the basis of the study by Giannoulaki et al. [62] was included (e.g., bathymetry, surface temperature, salinity, distance from the coast). All the data were downloaded using the Sdmpredictors package [63] from the databases WordClim [64] and Bio-Oracle [65]. An observation intensity map (see Figure S1) was generated based on the average number of monthly visits by OMMAG observers in each 10 km^2^ zone defined around Guadeloupe, allowing for the generation of pseudo-absence data. Distribution models were produced using an ensemble modeling approach, following Araújo and New [66], with the biomod2 package [67], after removing collinear variables. Six different methods were employed: Gradient Boosting Models [68], Random Forest [69], Generalized Linear Models [70], Classification Tree Analysis [71], Multivariate Adaptive Regression Splines [72], and Maximum Entropy [73]. These individual models were then aggregated into global models based on their performance values. The effectiveness of these global models was evaluated using the Receiver Operating Characteristic (ROC) metric [74]. The modeling method is detailed in Appendix A. All statistical analyses and graphs were produced using the free software R version 4.3.0.

### 2.4. Maritime Traffic Characterization

To study the exposure of each morphotype to maritime traffic, we took advantage of a previous study [44,45] that computed maritime traffic in the Guadeloupe archipelago from AIS data. Maritime traffic is significant in Guadeloupe, and Madon et al. [44] computed the density of maritime trajectories, calculated as the sum of distances traveled by all ships in a unit area [45], in the Guadeloupe archipelago. In order to identify the hotspot of maximum exposure of the two ecotypes of *T. truncatus* to maritime traffic, the predictions of occurrence obtained by the ensemble models for each morphotype and the density of shipping paths were standardized. These values were then multiplied to obtain a high product (close to 1) for geographical areas with both a high probability of occurrence and intense maritime traffic.

## 3. Results

This subsection will first focus on the results of photo-identification to describe the two morphotypes, their estimated abundances, and their distinguishing features. It will then move on to genetic analyses within Guadeloupe and comparisons to broader geographic scales. Finally, it will present the results of habitat modeling and identify hotspots for each morphotype in relation to maritime traffic.

### 3.1. Two Distinct Morphotypes of Different Estimated Abundance Identified Through Photo-Identification

**Figure 1:**
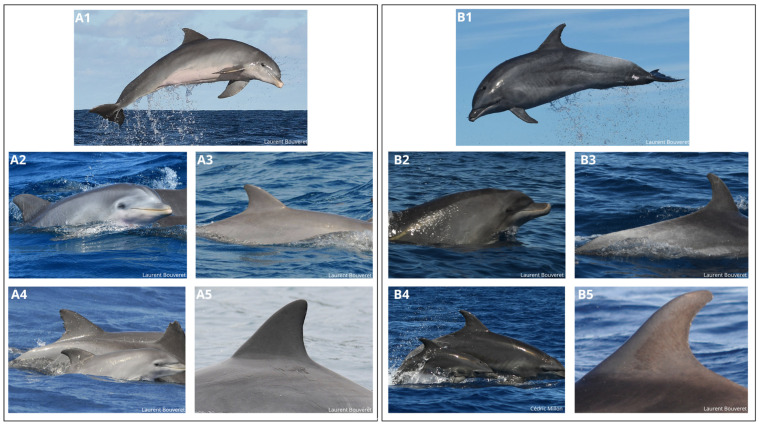
Panels of photos highlighting the morphological differences between the coastal morphotype (left-hand box, (**A1**–**A5**)) and the oceanic morphotype (right-hand box, (**B1**–**B5**)). 1: general view, B: anterior view, 3: lateral view of back, 4: mother and calf pair, 5: dorsal fin.

A total of 13,235 photographs and 72 videos of *T. truncatus* were taken by the OMMAG members between 30 September 2012 and 2 February 2022. Their analysis allowed the identification of two morphotypes of *T. truncatus*. These morphotypes have been called coastal and oceanic, due to their resemblance to coastal and oceanic ecotypes. The main differences between the two morphotypes are that the coastal morphotype is smaller, its coat is generally lighter, and it presents a rounded melon and a longer beak. Its dorsal fin is also more triangular (shorter but wider; Figure 1 left-hand box). In contrast, the oceanic morphotype, also called locally whitebacks, has a larger size associated with a darker coat, except at the back of the dorsal fin, where adults can often be seen to have a “white back” due to an accumulation of social markings (Figure 1). The dorsal fin of the oceanic morphotype is more falciform and elongated than that of the coastal (Figure 1). It can be noted that some oceanic dolphins present white patches of depigmentation (see Figure S2), usually near the caudal peduncle (Figure 1), which are never seen in coastal ones. Such pale skin patches in *T. truncatus* may appear during recovery from previous trauma [75] or may be a healing stage of pale dermatitis [76,77].

Twenty-seven coastal dolphins were individually identified and regularly captured over the monitoring period. They were observed repeatedly in particular areas, such as around the islets of Petite Terre and in the bays of the leeward coast located, respectively, southeast and west of the main Guadeloupe island. Concerning the oceanic morphotype, 165 individuals were identified. Among them, 43% were observed more than once between 2012 and 2022 and were captured between 2 and 4 times [78]. Two individuals were identified 10 times between 2012 and 2022. Spot observations, in which the apparent majority of individuals in a group were photographed in a single shot, made it possible to estimate the proportion of marked individuals, who appeared to represent at least 30% of the total number of individuals. According to the formula [79]:

$$N_{total}=\frac{N}{\theta },\;$$

with *N_total_* the estimated population size, *N* the calculated number of marked individuals, and θ′ the estimated proportion of marked individuals in the population, we hypothesized that at least 550 oceanic individuals may have visited the Guadeloupe in the last 15 years.

### 3.2. Mitochondrial DNA Analysis

#### 3.2.1. MCR Polymorphisms in the Guadeloupean Sample 

**Figure 2:**
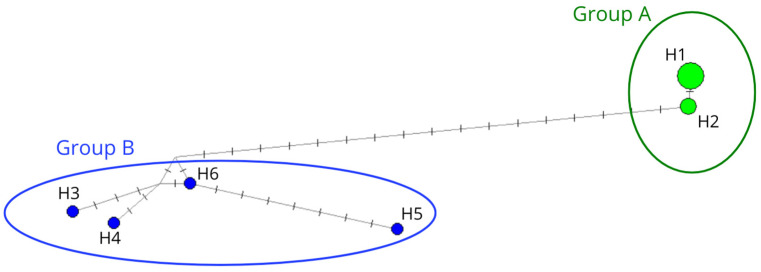
Median-joining network constructed with the 11 individual MCR sequences obtained in this study. The two groups of haplotypes (Groups A and B) are represented in the figure and differ by at least 19 mutations.

Six different haplotypes were determined from the 11 individuals sampled. All the sequence data are deposited in GenBank (Genbank accession: PQ082715-PQ082725). A median-joining network discriminated between two distinct groups of haplotypes (Groups A and B) differing by at least 19 mutations (Figure 2).

Group A comprised 2 haplotypes, determined from 7 individuals and differing at a single position corresponding to a heteroplasmic site. Group B had 4 haplotypes, each corresponding to a single individual. Groups A and B shared no common haplotype and differed by 19 to 21 mutations (Figure 2).

Both haplotype and nucleotide diversities were markedly lower for Group A (*Hd*_A_ = 0.476, *π_A_* = 0.019) than for Group B (*Hd_B_* = 1.000, *π_B_* = 0.261).

#### 3.2.2. Comparison with the Caribbean Dataset

**Figure 3:**
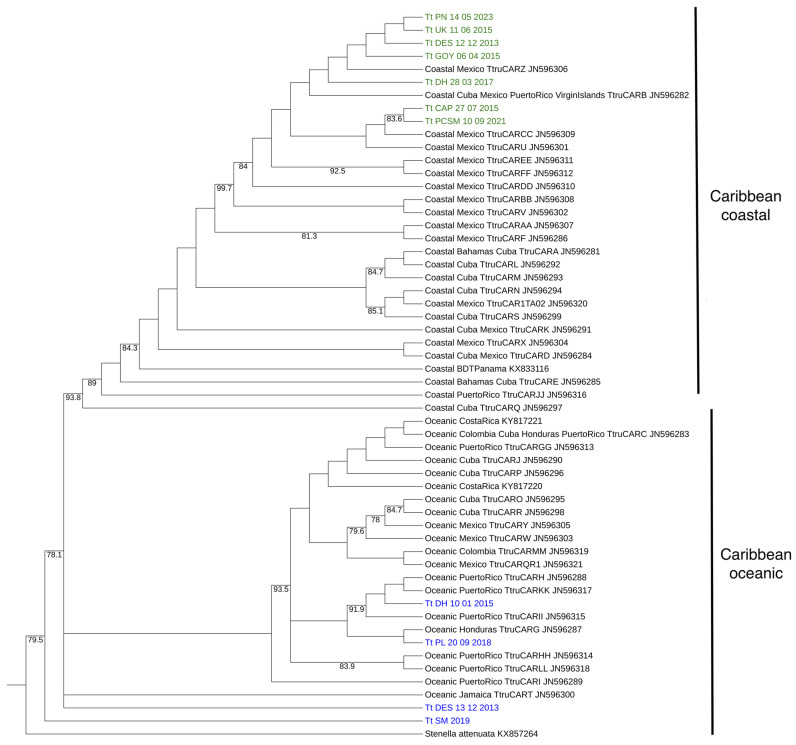
Phylogenetic tree constructed according to maximum similarity using the HKY model with 1000 bootstrap replicates. It presents the haplotypes from the Caribbean dataset and the sequences obtained in this study (in green the sequences from Group A, in blue those from Group B). Bootstrap values greater than 70% are indicated on the branches.

The Caribbean dataset (comprising 44 haplotypes identified by others on *T. truncatus* sampled from the Caribbean, sharing a common 288 bp segment determined in this study) was analyzed by constructing phylogenetic trees (Figure 3). The HKY substitution model was identified as the most suitable for this dataset. Oceanic and coastal Caribbean *T. truncatus* formed two distinct clades whose segregation was supported by a high bootstrap value (78.1). Coastal and oceanic Caribbean ecotype groups shown in Figure 3 had very similar haplotype and nucleotide diversity values (*Hd*_coastal_ = 0.836, *π*_coastal_ = 0.094; *Hd*_oceanic_ = 0.824, *π*_oceanic_ = 0.096). Fu’s *Fs* of Caribbean oceanic haplotypes showed a significant value (*Fs* = −5.727, *p*-value = 0.018). Caribbean coastal and oceanic ecotypes were significantly differentiated (*Fst* = 0.170, *Φst* = 0.748, and *Snn* = 1, *p*-value = 0.000 for all three indexes). This marked differentiation is reflected in the two groups of haplotypes determined from the Guadeloupean samples: all sequences in the Guadeloupean Group A (in green in Figure 3) grouped with Caribbean coastal haplotypes, while all sequences in Guadeloupean Group B (in blue in Figure 3) grouped with oceanic haplotypes.

#### 3.2.3. Comparison with the Atlantic Dataset

**Figure 4:**
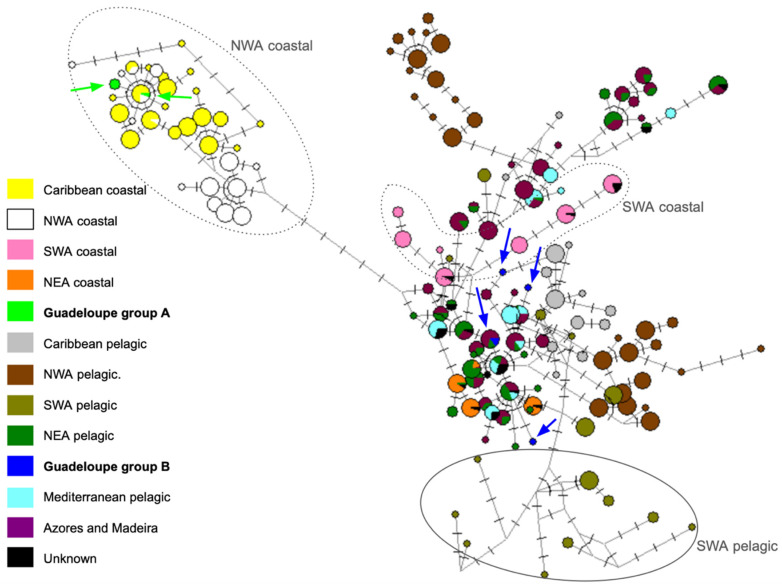
Haplotype network constructed from the “Atlantic” dataset and the data obtained in this study (indicated with arrows). The color code is shown in the figure. Each cross line symbolizes a mutation.

The extended dataset “Atlantic”, added to the Caribbean dataset, includes 287 haplotypes identified by others from *T. truncatus* sampled in the Atlantic Ocean. This Atlantic dataset was analyzed by constructing a haplotype network (Figure 4). *T. truncatus* from the Caribbean coast and coastal NWA formed a group isolated from all other haplotypes (separated by at least 8 mutations). Group A haplotypes from Guadeloupe (in green) belong to this particular group and share a haplotype with *T. truncatus* from the Caribbean coast (in yellow). Group B haplotypes from Guadeloupe (in dark blue) were located on the network close to oceanic haplotypes from the NEA, the Mediterranean, and the Azores. One haplotype was shared between individuals from the Azores and Madeira (in purple), oceanics from the NEA (in dark green), and a Guadeloupean Group B sequence (Tt_SM_2019).

### 3.3. Preferential Habitat Analysis

A total of *n* = 534 georeferenced photographs of the two morphotypes were used to compare the preferential habitats of the two morphotypes and estimate the differences between them. Three hundred eighty-four photographs concerned the coastal morphotype, and 150 the oceanic one. The overlap between the distribution patterns of the two morphotypes was estimated at 22.5% (with correction, which restores the density of presence of each morphotype as a function of the prevalence of environments in their range), indicating that they overlap over certain parts of the environmental space. The distribution patterns compared using permutation tests [61] indicated that the distribution patterns of the two morphotypes were similar but not interchangeable.

**Figure 5:**
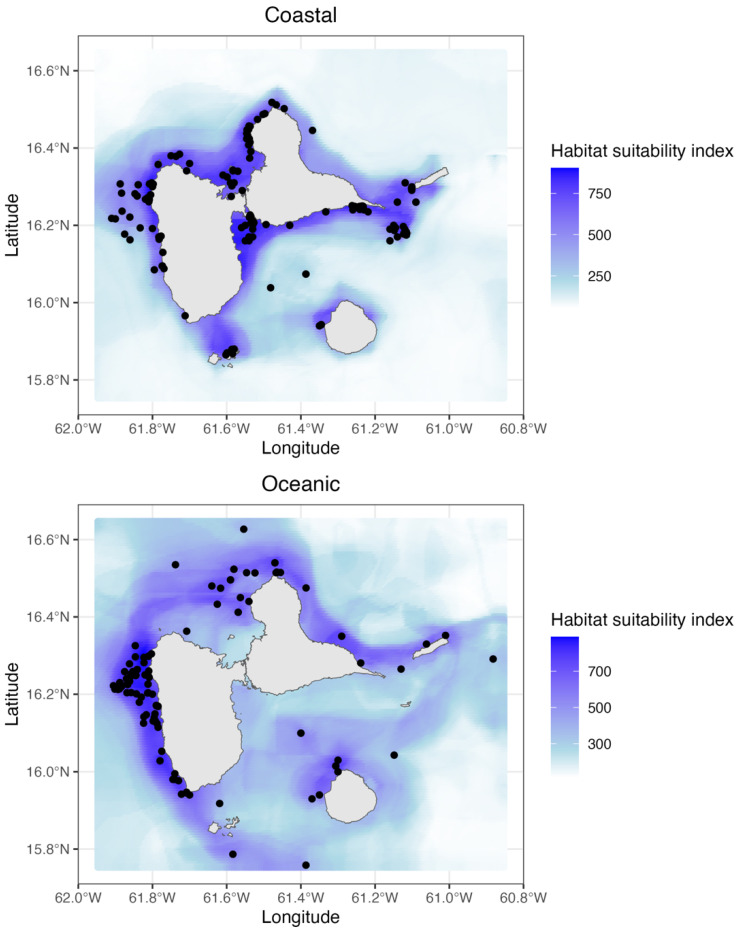
Habitat suitability maps for coastal and oceanic morphotypes of *T. truncatus*. (**Top panel**): Habitat suitability for the coastal morphotype. (**Bottom panel**): Habitat suitability for the oceanic morphotype. Color scale: The “Habitat suitability index” ranges from 0 (white) to 1000 (dark blue), where higher values indicate a greater probability of occurrence predicted by the model. Black dots: Observation data (sightings of the respective morphotype).

Species distribution models further estimated the distribution of the two morphotypes around the coasts of Guadeloupe. The evaluation values obtained validated the overall model established for each morphotype (ROC = 0.907 for coastal and ROC = 0.975 for oceanic). Models showed that both morphotypes of *T. truncatus* were distributed all around Guadeloupe, but that oceanic morphotype was mainly found further offshore than the coastal one (Figure 5). Maximum prediction values (close to 1000) for coastal morphotypes were found in the Northwest and Southeast of Guadeloupe, while those for oceanic ones extended all along the West coast and North of the island.

### 3.4. Exposure to Maritime Traffic 

**Figure 6:**
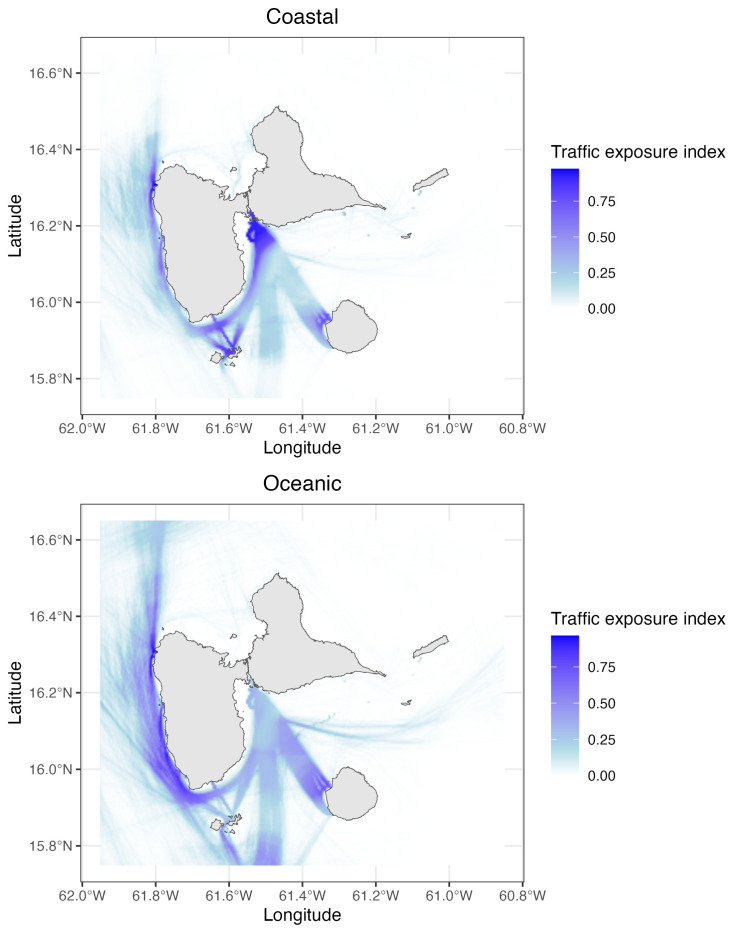
Hotspots of exposure to maritime traffic for coastal and oceanic morphotypes of *T. truncatus*. (**Top panel**): Exposure hotspots for the coastal morphotype. (**Bottom panel**): Exposure hotspots for the oceanic morphotype. Color scale: The Traffic exposure index corresponds to the product of standardized vessel trajectory lengths. Values range from 0 (white, low exposure) to 0.75 and above (dark blue, high exposure).

Maps indicating maritime traffic exposure hotspots revealed strongly different situations for the two morphotypes (Figure 6). Hotspots of exposition for oceanic morphotype were detected along the West coast of the island, which corresponds mainly to international shipping [44,45]. Coastal morphotype appeared more exposed in the Petit Cul-de-Sac Marin (the bay separating the two Southern parts of the island of Guadeloupe) but also in Marie-Galante and Les Saintes islands, corresponding to the transit zone towards the port area of the island [44]. The coastal morphotype also appeared highly exposed on the routes linking the islands of the archipelago, which corresponds to the regular inter-island ferry routes, especially as this traffic is carried out by fast vessels [44].

These results emphasize the significant variation in exposure risks between the two morphotypes, highlighting the need for tailored conservation measures.

## 4. Discussion

The occurrence of the two ecotypes of *T. truncatus*, coastal and oceanic, has been documented across a wide geographical range. However, the situation is not uniform and appears to be more complex to decipher in certain geographical areas such as the Azores and Madeira [35], the Pacific Ocean, or the North-East Atlantic (NEA) [3,33,80]. The three criteria defined by Stronen et al. [10] (i.e., phenotypic variation, differences in preferred habitats, and genetic differentiation) provide a solid basis for recognizing and characterizing the presence of both ecotypes in a given location. Even if this basis for ecotype identification is not standardized across studies, with some relying solely on ecological and morphological parameters [20] or genetic analyses (e.g., [41]), the failure to detect both ecotypes in some places seems to be linked more to a real heterogeneity in their distribution than to technical issues of identification.

Here, we sought to determine whether the two morphotypes of *T. truncatus* recently detected by citizen science observers around the Guadeloupe archipelago met the three criteria of Stronen et al. [10] and could therefore be considered coastal and oceanic eco-types, whose existence would thus be proven locally for the first time.

### 4.1. Two Distinct Morphotypes with Different Preferred Habitats

Photo-analyses revealed the presence of two distinct morphotypes of *T. truncatus* in Guadeloupe, the coastal morphotype and the oceanic morphotype, which are clearly evocative of the general morphologies of the two Atlantic ecotypes [1,28,34].

Analysis of spatial distribution patterns showed that the two morphotypes in Guadeloupe are distributed in space in a similar but not identical way, which is generally expected for closely related taxonomic groups [61]. The results of the habitat modeling confirmed these findings and specified the preferred habitats of each morphotype. The preferred habitat of the coastal morphotype was found to be mainly near the coast, while that of the oceanic morphotype was distributed further offshore.

In the Atlantic, significant habitat differences between the two ecotypes of *T. truncatus* are well recognized. The coastal ecotype was mainly concentrated in areas of lower bathymetry, whereas the oceanic ecotype had a wider spatial distribution associated with a more flexible use of its habitat (e.g., [34,81]). Areas of partial overlap between coastal and oceanic ecotypes in NWA and SWA have also been demonstrated (e.g., [34,82]), similar to the results of this study. In the Gulf of Mexico, where such overlap exists, it has been hypothesized that the distribution of the oceanic ecotype in both neritic and oceanic habitats could be one of the reasons for this overlap [36].

In the marine environment, local variations in habitat characteristics can drive the development of specialized ecological niches [29]. In cetaceans, ecological niches appear to be linked to water temperature and depth, as well as any factors affecting the distribution and abundance of their prey [37,81]. Indeed, habitat differences appear to influence the feeding behaviour of *T. truncatus* [31,83,84]. In NWA, it was suggested that some of the differences between the two ecotypes were probably associated with distinct feeding ecologies directly related to their habitat [1]. More broadly, ecological opportunities for specialization may have been the main driver of ecological, morphological, and genetic divergence in *T. truncatus* [81]. 

### 4.2. Two Distinct Guadeloupean Genetic Groups of T. truncatus Correlated to Caribbean Ecotypes

Analysis of the polymorphisms in the mitochondrial DNA of *T. truncatus* in Guadeloupe identified two distinct genetic groups strongly separated by at least 19 nucleotide differences out of 676 bp.

When compared to larger geographic scale DNA sequence data (i.e., Caribbean data, and then whole Atlantic data), these two groups presented obvious sequence similarities with the coastal *T. truncatus* ecotype in the case of one, and with the oceanic ecotype in the case of the other.

It can be noted that similar results were obtained by Rodriguez-Ferrer et al. [85] in Puerto Rico.

In Guadeloupe, the haplotype diversity and nucleotide diversity were markedly different between the two groups. The coastal group (Group A) exhibited lower haplotype diversity (HdA = 0.476) and nucleotide diversity (πA = 0.019) compared to the oceanic group (Group B), which showed a haplotype diversity (HdB = 1.000) and nucleotide diversity (πB = 0.261) values much higher. Although this observation should be interpreted in the light of the small number of samples (*n* = 7 and *n* = 4, respectively), these values were similar to those determined in groups of coastal *T. truncatus* in the NWA [1], SWA [2], and NEA [3]. The observed differences in genetic diversity between the coastal and oceanic ecotypes in Guadeloupe are likely shaped by historical demographic events, ecological factors such as habitat size and connectivity, and their respective mobility and gene flow.

Differentiation indices between the two ecotypes in Caribbean *T. truncatus*, including the new data from Guadeloupe, presented high values reflecting very strong genetic differentiations between the two ecotypes. The *F_ST_* and *Φ_ST_* values determined were very close to those calculated by Costa et al. [1] between the two ecotypes in the NWA (this study, *F_ST_* = 0.170 and *Φ_ST_* = 0.748; Costa et al. [1], *F_ST_* = 0.21 and *Φ_ST_* = 0.71).

For oceanic ecotypes, geographical proximity seems to have less influence on genetic closeness, and Guadeloupean oceanic *T. truncatus* appears to be as close to the Caribbean oceanics as they are to those from the Mediterranean and the NEA. Generally speaking, in the Atlantic, oceanic *T. truncatus* appear to form a single large genetic group, supporting the designation of the oceanic ecotype of *T. truncatus* as ‘Worldwide distributed form’ (WDF), proposed by Tezanos-Pinto et al. [80]. Similar results have recently been highlighted by Gómez-Lobo et al. [86], who demonstrated high connectivity with the Northeast Atlantic via mitochondrial variation in *T. truncatus* from the Canary Islands.

The Atlantic basin-scale network highlighted that the genetic divergence between the two ecotypes of *T. truncatus* was much more marked in the Northwest Atlantic than in the Northeast Atlantic, corresponding clearly to an older separation. This finding is consistent with the results of Louis et al. [4], who showed that the oldest divergence between oceanic and coastal ecotypes occurred in the Northwest Atlantic (estimated at 80,000 BP) and the most recent in the Northeast Atlantic (estimated at 12,000 BP). This information supports the hypothesis that coastal Caribbean *T. truncatus* emerged from Atlantic oceanic ones around 80,000 years ago [4]. This emergence is likely to have occurred under the influence of genetic drift and possible reproductive isolation, leading to the strong genetic differentiation observed nowadays between the two ecotypes in the Northwest Atlantic. Whether they are on different evolutionary trajectories remains to be seen.

### 4.3. Integration of Genetic, Habitat, and Morphological Data to Distinguish Two Distinct Ecotypes with Different Management Implications

The integration of genetic, habitat, and morphological data (Table S4) provides a robust framework for identifying two distinct ecotypes [10] of *T. truncatus* within the Guadeloupe Archipelago. These three independent lines of evidence—distinct genetic groups, different morphologies, and their separate habitat use—allowed us to distinguish the morphotypes of *T. truncatus* in Guadeloupe as two distinct ecotypes.

Genetic analysis revealed two distinct genetic groups in Guadeloupe: the coastal ecotype exhibited low genetic diversity, high site fidelity, and apparent genetic isolation from neighboring populations, while the oceanic ecotype showed higher genetic diversity and presumably greater gene flow. Habitat modeling corroborated these findings by demonstrating that the two morphotypes do not occupy the same habitats. The coastal morphotype is primarily found in nearshore areas, whereas the oceanic morphotype prefers deeper, offshore habitats, highlighting their different ecological requirements. Morphological differences between the two groups—such as body size, fin shape, and coloration—further distinguish them and are likely to be linked to their habitat preferences and feeding behaviors. Together, these independent lines of evidence confirmed the ecotype status of *T. truncatus* in Guadeloupe, emphasizing the need for targeted conservation efforts that address the specific requirements and threats facing each ecotype.

The marked differentiation and the probable limited gene flow between the two sympatric *T. truncatus* ecotypes around Guadeloupe lead to concerns in terms of conservation, especially about possible differences of interactions with human activities and the need for tailored management strategies. Coastal populations generally exhibit a higher level of site fidelity, low abundance, and are genetically isolated from other neighboring populations (e.g., [37,41,87]). Because of their adaptations to local conditions, habitat degradation could further affect coastal populations, which are vulnerable to rapid changes in their habitat, particularly those caused by human activities (e.g., [88]). In Guadeloupe, the coastal ecotype has a very small population (around 30 individuals, according to photo-identification data), which raises questions about the impact of anthropogenic activities in terms of disturbance or pollution [89]. This ecotype’s dependence on specific habitats, such as the Petit Cul-de-Sac Marin, highlights the need for protective zoning measures, such as time-area restrictions to reduce disturbance from maritime traffic, as well as actions to limit pollution and noise in these critical habitats. Long-term monitoring of population trends and genetic diversity is essential to ensure the resilience of this ecotype.

The oceanic ecotype raises fewer concerns *a priori* because of its belonging to a WDF, because of its greater abundance (550 different individuals estimated by photo-identification recapture in the Guadeloupean waters), and because of its suspected greater adaptability. But it could be exposed to specific risks at local to regional scales. It is, for instance, more exposed to accidental capture by fishing gear and even targeted whaling in Saint Vincent and the Grenadines [90].

The environmental impacts of maritime traffic, including specifically on cetaceans, are well documented (e.g., [44,91,92]). Several studies have reported short-term behavioral responses of *T. truncatus* exposed to shipping traffic, such as an increase in their dive time [93] or changes in their breathing and surfacing patterns [94,95]. Allen et al. [96] showed in Florida that *T. truncatus* decreased their use of feeding sites during periods of high maritime traffic density. Russell et al. [97] defined ‘high-risk management zones’ as geographical areas where a high density of cetaceans and intense maritime traffic converge. In view of the results obtained in this study, the two ecotypes of *T. truncatus* in Guadeloupe are exposed to intense maritime traffic in at least part of their habitats. The oceanic ecotype appears to have more hot spots of exposure, which may be explained by its wider ecological niche. The coastal ecotype is most exposed in the Petit Cul-de-Sac Marin, which is a compulsory passage zone for all ships transiting to the island’s port area. Generally speaking, in Guadeloupe, *T. truncatus* are particularly exposed to maritime traffic in the Côte-sous-le-vent (the west coast of the island), which could be considered a ‘high-risk management zone’ for the species.

Our findings underscore the importance of tailoring conservation strategies to the distinct needs of these ecotypes. The coastal ecotype, with its small and localized population, requires intensive habitat protection and disturbance mitigation, while the oceanic ecotype would benefit from regional collaborations addressing broader-scale threats. By identifying these management priorities, this study contributes to the development of evidence-based conservation strategies for *T. truncatus* in Guadeloupe.

## 5. Conclusions

We obtained positive results for all three criteria: morphological differences between the two morphotypes), genetic differentiation (two distinct genetic groups along the coasts of Guadeloupe, corresponding to previously published coastal and oceanic ecotype genetic differences), and habitat differences (distribution patterns not interchangeable, and oceanic *T. truncatus* found further offshore in terms of suitable habitats). This triple confirmation supports the ecotype status of the coastal and oceanic morphotypes of *T. truncatus* in the Guadeloupe archipelago.

Guadeloupean waters are part of the Agoa sanctuary, specifically dedicated to marine mammals, where the *T. truncatus* is an emblematic and heritage species of the archipelago. The two ecotypes of *T. truncatus* identified here exhibit clear distinctions in morphology, genetics, and habitat use. Further studies, including, for example, ecological analyses to assess possible differences in diet, foraging strategies, and ecotoxicological exposure, could enhance our knowledge about their possible differential exposure to human activities. Based on the results presented here, we propose considering the two ecotypes of *T. truncatus* as distinct management units within the same species in Guadeloupe. 

## Data Availability

All the sequence data are deposited in the GenBank (Genbank accession: PQ082715-PQ082725).

## Supplementary Materials

The following supporting information can be downloaded at: https://www.mdpi.com/article/10.3390/ani15010108/s1, Figure S1: OMMAG observation intensity map used to approximate the sampling effort associated with observation data for the two morphotypes of *T. truncatus* in the Guadeloupe archipelago. Figure S2: Examples of depigmentation spots observed on oceanic dolphins (photographs: Laurent Bouveret, OMMAG). Table S1: Summary of distinction criteria used to differentiate *T. truncatus* ecotypes in the different oceanic basins; Table S2: List of haplotypes used for the Caribbean dataset (accession numbers are associated); Table S3: List of haplotypes used for the Atlantic dataset (accession numbers are associated). Table S4: Environmental variables used for habitat modeling and their ecological relevance. Table S5: Summary of genetic diversity indices, habitat overlaps and morphological differences between coastal and oceanic morphotypes of *T. truncatus* in the Guadeloupe archipelago.

## Appendix A

The GPS coordinates of the observations of the two morphotypes of *T. truncatus* collected by OMMAG were recorded. These are presence data only. For oceanics, GPS data for each sighting, associated with the confirmation of the morphotype via photo-identification, had already been collected. For the coastal morphotype, data were extracted from the Kakila database [43], which compiles the presence data, acquired through participatory science, of cetaceans in the waters of the Guadeloupe archipelago. Data on the presence of the two morphotypes were specifically targeted for the period 2012 to 2022. The OMMAG observation pressure map, presented in Figure S1, was used to approximate the sampling effort. All statistical analyses and graphs were produced using the free software R version 4.3.0.

Environmental data were downloaded from the Sdmpredictors package [63], provides access to several georeferenced databases of environmental variables. Several bioclimatic covariates and covariates associated with the hydrology of water bodies from the WordClim [64] and Bio-Oracle [65] databases, respectively, were thus extracted for the entire study area (GPS coordinates: 15.5, 16.9; −60.5, −62.5). The environmental variables were selected based on the study by Giannoulaki et al. [62] and included (Table S5): mean bathymetry, mean, minimum, and maximum chlorophyll a concentration, mean, minimum, and maximum surface temperature, mean salinity, distance from the coast, bottom slope, light attenuation coefficient and photosynthetically active radiation (PAR, a proxy for water turbidity).

The aim was to model the ecological niches of the two morphotypes using an ensemble model [66], an approach adapted to the case of presence data only [98]. The opportunistic nature of the data meant that a series of pseudo-absences had to be set up to mimic the absence of the morphotypes within the study area. To do this, as many weighted random pseudo-absence data as occurrence data were generated from the sampling effort data. For each morphotype, 5 sets of pseudo-absences were established to assess the uncertainty of the predictions. As high collinearity between model variables can lead to errors in parameter estimation, collinearity was assessed by calculating the VIF (variance inflation factor) for each variable. Variables with a VIF greater than 10 were removed from the environmental dataset due to high collinearity.

The ecological niche of each morphotype was modelled using biomod2 [67], which allows several modelling approaches to be implemented simultaneously. Six different methods were used: Gradient Boosting Models (GBM), Random Forest (RF), Generalized Linear Models (GLM), Classification Tree Analysis (CTA), Multivariate Adaptive Regression Splines (MARS), and Maximum Entropy (MAXENT). The overall model was established by aggregating the different models based on their individual performance values. The models produced were evaluated using the Receiver Operating Characteristic (ROC) metric, which summarizes a model’s classification performance. Each model was tested using cross-validation, which allows several validation sets to be drawn from the same database to test the model’s validation performance. Each final ensemble model was tested on a subset comprising 30% of the original dataset. The projections of the ensemble models were used to obtain a map defining the predictions of the occurrences of each morphotype, to which uncertainty maps were added, providing information on the levels of reliability of the predictions made.

## References

[1] Costa A.P.B., Mcfee W., Wilcox L.A., Archer F.I., Rosel P.E. (2022). The Common Bottlenose Dolphin (*Tursiops truncatus*) Ecotypes of the Western North Atlantic Revisited: An Integrative Taxonomic Investigation Supports the Presence of Distinct Species. Zool. J. Linn. Soc..

[2] Fruet P., Secchi E., Tullio J.D.D., Simões-Lopes P.C., Daura-Jorge F., Costa A.P.B., Vermeulen E., Flores P., Genoves R., Laporta P. (2017). Genetic Divergence between Two Phenotypically Distinct Bottlenose Dolphin Ecotypes Suggests Separate Evolutionary Trajectories. Ecol. Evol..

[3] Louis M., Viricel A., Lucas T., Peltier H., Alfonsi E., Berrow S., Brownlow A., Covelo P., Dabin W., Deaville R. (2014). Habitat-driven Population Structure of Bottlenose Dolphins, *Tursiops truncatus*, in the North-East Atlantic. Mol. Ecol..

[4] Louis M., Galimberti M., Archer F., Berrow S., Brownlow A., Fallon R., Nykänen M., O’Brien J., Roberston K.M., Rosel P.E. (2021). Selection on Ancestral Genetic Variation Fuels Repeated Ecotype Formation in Bottlenose Dolphins. Sci. Adv..

[5] Andrews K.R., Epstein B., Leslie M.S., Fiedler P., Morin P.A., Hoelzel A.R. (2021). Genomic Signatures of Divergent Selection Are Associated with Social Behaviour for Spinner Dolphin Ecotypes. Mol. Ecol..

[6] Jung J.-L. (2017). Approches Moléculaires Pour L’étude de la Biodiversité des Mammifères Marins. Habilitation Thesis.

[7] Richard G., Titova O.V., Fedutin I.D., Steel D., Meschersky I.G., Hautin M., Burdin A.M., Hoyt E., Filatova O.A., Jung J.-L. (2018). Cultural Transmission of Fine-Scale Fidelity to Feeding Sites May Shape Humpback Whale Genetic Diversity in Russian Pacific Waters. J. Hered..

[8] Whitehead H., Laland K.N., Rendell L., Thorogood R., Whiten A. (2019). The Reach of Gene–Culture Coevolution in Animals. Nat. Commun..

[9] Turesson G. (1922). The Genotypical Response of the Plant Species to the Habitat. Hereditas.

[10] Stronen A.V., Norman A.J., Vander Wal E., Paquet P.C. (2022). The Relevance of Genetic Structure in Ecotype Designation and Conservation Management. Evol. Appl..

[11] Lowry D.B. (2012). Ecotypes and the Controversy over Stages in the Formation of New Species: STAGES IN SPECIATION. Biol. J. Linn. Soc..

[12] Bruyn P.J.N., Tosh C.A., Terauds A. (2013). Killer Whale Ecotypes: Is There a Global Model?. Biol. Rev. Camb. Philos. Soc..

[13] Taylor B.L., Archer F.I., Martien K.K., Rosel P.E., Hancock-Hanser B.L., Lang A.R., Leslie M.S., Mesnick S.L., Morin P.A., Pease V.L. (2017). Guidelines and Quantitative Standards to Improve Consistency in Cetacean Subspecies and Species Delimitation Relying on Molecular Genetic Data. Mar. Mammal Sci..

[14] Morin P.A., McCarthy M.L., Fung C.W., Durban J.W., Parsons K.M., Perrin W.F., Taylor B.L., Jefferson T.A., Archer F.I. (2024). Revised Taxonomy of Eastern North Pacific Killer Whales (*Orcinus orca*): Bigg’s and Resident Ecotypes Deserve Species Status. R. Soc. Open Sci..

[15] Castelblanco-Martínez D.N., Slone D.H., Landeo-Yauri S.S., Ramos E.A., Alvarez-Alemán A., Attademo F.L.N., Beck C.A., Bonde R.K., Butler S.M., Cabrias-Contreras L.J. (2021). Analysis of Body Condition Indices Reveals Different Ecotypes of the Antillean Manatee. Sci. Rep..

[16] Committee on Taxobomy List of Marine Mammal Species and Subspecies. http://marinemammalscience.org.

[17] Rosel P.E., Taylor B.L., Hancock-Hanser B.L., Morin P.A., Archer F.I., Lang A.R., Mesnick S.L., Pease V.L., Perrin W.F., Robertson K.M. (2017). A Review of Molecular Genetic Markers and Analytical Approaches That Have Been Used for Delimiting Marine Mammal Subspecies and Species. Mar. Mammal Sci..

[18] Moura A.E., Nielsen S.C.A., Vilstrup J.T., Moreno-Mayar J.V., Gilbert M.T.P., Gray H.W.I., Natoli A., Möller L., Hoelzel A.R. (2013). Recent Diversification of a Marine Genus (*Tursiops* spp.) Tracks Habitat Preference and Environmental Change. Syst. Biol..

[19] Wells R.S., Natoli A., Braulik G. (2019). Tursiops truncatus. The IUCN Red List of Threatened Species 2019: E.T22563A156932432. IUCN Red List of Threatened Species.

[20] Vollmer N.L., Rosel P.E. (2013). A Review of Common Bottlenose Dolphins (*Tursiops Truncatus Truncatus*) in the Northern Gulf of Mexico: Population Biology, Potential Threats, and Management. Southeast. Nat..

[21] Costa A.P.B., Loch C., Simões-Lopes P.C. (2016). Variations and Anomalies in the Vertebral Column of the Bottlenose Dolphin (*Tursiops Truncatus*) from Southern Brazil. Lat. Am. J. Aquat. Mamm..

[22] Costa A.P.B., Archer F.I., Rosel P.E., Perrin W.F. (2023). *Tursiops Truncatus Nuuanu*, a New Subspecies of the Common Bottlenose Dolphin from the Eastern Tropical Pacific. J. Mamm. Evol..

[23] Alexandre B.G., Cruz M.M., do Amaral K.B., Hoffmann L.S., de Freitas T.R.O., Zanini R. (2024). Exploring mtDNA Databases to Evaluate the Population Structure and Genetic Diversity of *Tursiops Truncatus* in the Atlantic Ocean: Implications for the Conservation of a Small, Offshore Population. Ecologies.

[24] Lowther-Thieleking J.L., Archer F.I., Lang A.R., Weller D.W. (2015). Genetic Differentiation among Coastal and Offshore Common Bottlenose Dolphins, *Tursiops Truncatus*, in the Eastern North Pacific Ocean. Mar. Mammal Sci..

[25] Särnblad A., Danbolt M., Dalén L., Amir O.A., Berggren P. (2011). Phylogenetic Placement and Population Structure of Indo-Pacific Bottlenose Dolphins (*Tursiops Aduncus*) off Zanzibar, Tanzania, Based on mtDNA Sequences. Mar. Mammal Sci..

[26] Duffield D.A., Ridgway S.H., Cornell L.H. (1983). Hematology Distinguishes Coastal and Offshore Forms of Dolphins (*Tursiops*). Can. J. Zool..

[27] Mead J.G., Potter C.W., Leatherwood S., Reeves R.R. (1990). Natural History of Bottlenose Dolphins along the Central Atlantic Coast of the United States. The Bottlenose Dolphin.

[28] Mead J.G., Potter C.W. (1995). Recognizing Two Populations of the Bottlenose Dolphin (*Tursiops truncatus*) of the Atlantic Coast of North America-Morphologic and Ecologic Considerations. Int. Mar. Biol. Res. Inst. IBI Rep..

[29] Hoelzel A.R., Potter C.W., Best P.B. (1998). Genetic Differentiation between Parapatric ‘Nearshore’ and ‘Offshore’ Populations of the Bottlenose Dolphin. Proc. R. Soc. Lond. B.

[30] Kingston S.E., Rosel P.E. (2004). Genetic Differentiation among Recently Diverged Delphinid Taxa Determined Using AFLP Markers. J. Hered..

[31] Rosel P.E., Hansen L., Hohn A.A. (2009). Restricted Dispersal in a Continuously Distributed Marine Species: Common Bottlenose Dolphins *Tursiops truncatus* in Coastal Waters of the Western North Atlantic: Fine-scale population structure in *Tursiops*. Mol. Ecol..

[32] Costa A.P.B., Fruet P.F., Secchi E.R., Daura-Jorge F.G., Simões-Lopes P.C., Di Tullio J.C., Rosel P.E. (2021). Ecological Divergence and Speciation in Common Bottlenose Dolphins in the Western South Atlantic. J. Evol. Biol..

[33] Natoli A., Peddemors V.M., Rus Hoelzel A. (2004). Population Structure and Speciation in the Genus Tursiops Based on Microsatellite and Mitochondrial DNA Analyses. J. Evol. Biol..

[34] Simões-Lopes P., Daura-Jorge F., Lodi L., Bezamat C., Costa A., Wedekin L. (2019). Bottlenose Dolphin Ecotypes of the Western South Atlantic: The Puzzle of Dorsal Fin Shapes, Colors and Habitats. Aquat. Biol..

[35] Quérouil S., Silva M.A., Freitas L., Prieto R., Magalhães S., Dinis A., Alves F., Matos J.A., Mendonça D., Hammond P.S. (2007). High Gene Flow in Oceanic Bottlenose Dolphins (*Tursiops truncatus*) of the North Atlantic. Conserv. Genet..

[36] Caballero S., Islas-Villanueva V., Tezanos-Pinto G., Duchene S., Delgado-Estrella A., Sanchez-Okrucky R., Mignucci-Giannoni A.A. (2012). Phylogeography, Genetic Diversity and Population Structure of Common Bottlenose Dolphins in the Wider Caribbean Inferred from Analyses of Mitochondrial DNA Control Region Sequences and Microsatellite Loci: Conservation and Management Implications: Phylogeography of Bottlenose Dolphins in the Caribbean. Anim. Conserv..

[37] Barragán-Barrera D.C., May-Collado L.J., Tezanos-Pinto G., Islas-Villanueva V., Correa-Cárdenas C.A., Caballero S. (2017). High Genetic Structure and Low Mitochondrial Diversity in Bottlenose Dolphins of the Archipelago of Bocas Del Toro, Panama: A Population at Risk?. PLoS ONE.

[38] Fajardo D., Alejandra M. (2017). Genetic Diversity and Population Structure of the Common Bottlenose Dolphin (Tursiops truncatus) in Two Areas of the Colombian and Panamanian Caribbean Inferred from Mitochondrial Control Region.

[39] Gregor J.W. (1944). The Ecotype. Biol. Rev..

[40] Currey R.J.C., Dawson S.M., Slooten E. (2011). Tursiops truncatus Fiordland Subpopulation. The IUCN Red List of Threatened Species 2011: E. T194300A67107359. IUCN Red List of Threatened Species.

[41] Louis M., Buanic M., Lefeuvre C., Nilliot P.L., Ridoux V., Spitz J. (2017). Strong Bonds and Small Home Range in a Resident Bottlenose Dolphin Community in a Marine Protected Area (Brittany, France, Northeast Atlantic). Mar. Mammal Sci..

[42] Martinho F., Pereira A., Brito C., Gaspar R., Carvalho I. (2015). Structure and Abundance of Bottlenose Dolphins (*Tursiops truncatus*) in Coastal Setúbal Bay, Portugal. Mar. Biol. Res..

[43] Coché L., Arnaud E., Bouveret L., David R., Foulquier E., Gandilhon N., Jeannesson E., Le Bras Y., Lerigoleur E., Lopez P.J. (2021). Kakila Database: Towards a FAIR Community Approved Database of Cetacean Presence in the Waters of the Guadeloupe Archipelago, Based on Citizen Science. Biodivers. Data J..

[44] Madon B., Le Guyader D., Jung J.-L., De Montgolfier B., Lopez P.J., Foulquier E., Bouveret L., Le Berre I. (2022). Pairing AIS Data and Underwater Topography to Assess Maritime Traffic Pressures on Cetaceans: Case Study in the Guadeloupean Waters of the Agoa Sanctuary. Mar. Policy.

[45] Le Berre I., Foulquier E., Le Guyader D., Iphar C., Sahuquet M., Lopez P.J. (2024). De l’emprise à l’empreinte: Cartographier la donnée AIS pour qualifier l’occupation de l’espace maritime caribéen. Cybergeo Eur. J. Geogr..

[46] Garrigue C., Dodemont R., Steel D., Baker C. (2004). Organismal and ‘Gametic’ Capture-Recapture Using Microsatellite Genotyping Confirm Low Abundance and Reproductive Autonomy of Humpback Whales on the Wintering Ground of New Caledonia. Mar. Ecol.-Prog. Ser..

[47] Girardet J., Sarano F., Richard G., Tixier P., Guinet C., Alexander A., Sarano V., Vitry H., Preud’homme A., Heuzey R. (2022). Long Distance Runners in the Marine Realm: New Insights into Genetic Diversity, Kin Relationships and Social Fidelity of Indian Ocean Male Sperm Whales. Front. Mar. Sci..

[48] Kearse M., Moir R., Wilson A., Stones-Havas S., Cheung M., Sturrock S., Buxton S., Cooper A., Markowitz S., Duran C. (2012). Geneious Basic: An Integrated and Extendable Desktop Software Platform for the Organization and Analysis of Sequence Data. Bioinformatics.

[49] Altschul S.F., Madden T.L., Schäffer A.A., Zhang J., Zhang Z., Miller W., Lipman D.J. (1997). Gapped BLAST and PSI-BLAST: A New Generation of Protein Database Search Programs. Nucleic Acids Res..

[50] Vollmer N.L., Rosel P. (2017). Fine-Scale Population Structure of Common Bottlenose Dolphins (*Tursiops truncatus*) in Offshore and Coastal Waters of the US Gulf of Mexico. Mar. Biol..

[51] Excoffier L., Lischer H.E.L. (2010). Arlequin Suite Ver 3.5: A New Series of Programs to Perform Population Genetics Analyses under Linux and Windows. Mol. Ecol. Resour..

[52] Fu Y.X. (1997). Statistical Tests of Neutrality of Mutations against Population Growth, Hitchhiking and Background Selection. Genetics.

[53] Tajima F. (1989). The Effect of Change in Population Size on DNA Polymorphism. Genetics.

[54] Rozas J., Ferrer-Mata A., Sánchez-DelBarrio J.C., Guirao-Rico S., Librado P., Ramos-Onsins S.E., Sánchez-Gracia A. (2017). DnaSP 6: DNA Sequence Polymorphism Analysis of Large Data Sets. Mol. Biol. Evol..

[55] Hudson R.R. (2000). A New Statistic for Detecting Genetic Differentiation. Genetics.

[56] Tamura K., Stecher G., Kumar S. (2021). MEGA11: Molecular Evolutionary Genetics Analysis Version 11. Mol. Biol. Evol..

[57] Trifinopoulos J., Nguyen L.-T., von Haeseler A., Minh B.Q. (2016). W-IQ-TREE: A Fast Online Phylogenetic Tool for Maximum Likelihood Analysis. Nucleic Acids Res..

[58] Letunic I., Bork P. (2021). Interactive Tree Of Life (iTOL) v5: An Online Tool for Phylogenetic Tree Display and Annotation. Nucleic Acids Res..

[59] Schoener T.W. (1968). The Anolis Lizards of Bimini: Resource Partitioning in a Complex Fauna. Ecology.

[60] Di Cola V., Broennimann O., Petitpierre B., Breiner F.T., D’Amen M., Randin C., Engler R., Pottier J., Pio D., Dubuis A. (2017). Ecospat: An R Package to Support Spatial Analyses and Modeling of Species Niches and Distributions. Ecography.

[61] Warren D., Glor R., Turelli M. (2008). Environmental Niche Equivalency versus Conservatism: Quantitative Approaches to Niche Evolution. Evol. Int. J. Org. Evol..

[62] Giannoulaki M., Markoglou E., Valavanis V.D., Alexiadou P., Cucknell A., Frantzis A. (2017). Linking Small Pelagic Fish and Cetacean Distribution to Model Suitable Habitat for Coastal Dolphin Species, Delphinus Delphis and *Tursiops truncatus*, in the Greek Seas (Eastern Mediterranean). Aquat. Conserv. Mar. Freshw. Ecosyst..

[63] Bosch S., Tyberghein L., De Clerck O. (2018). Sdmpredictors: Species Distribution Modelling Predictor Datasets. R Package Version 0.2.

[64] Hijmans R.J., Cameron S.E., Parra J.L., Jones P.G., Jarvis A. (2005). Very High Resolution Interpolated Climate Surfaces for Global Land Areas. Int. J. Climatol..

[65] Tyberghein L., Verbruggen H., Pauly K., Troupin C., Mineur F., De Clerck O. (2012). Bio-ORACLE: A Global Environmental Dataset for Marine Species Distribution Modelling. Glob. Ecol. Biogeogr..

[66] Araújo M.B., New M. (2007). Ensemble Forecasting of Species Distributions. Trends Ecol. Evol..

[67] Thuiller W., Georges D., Engler R. (2014). Biomod2: Ensemble Platform for Species Distribution Modelling. https://cran.r-project.org/web/packages/biomod2/index.html.

[68] Natekin A., Knoll A. (2013). Gradient Boosting Machines, a Tutorial. Front. Neurorobot..

[69] Breiman L. (2001). Random Forests. Mach. Learn..

[70] Hastie T.J., Pregibon D. (2017). Generalized Linear Models. Statistical Models in S.

[71] Vayssières M.P., Plant R.E., Allen-Diaz B.H. (2000). Classification Trees: An Alternative Non-Parametric Approach for Predicting Species Distributions. J. Veg. Sci..

[72] Friedman J.H. (1991). Multivariate Adaptive Regression Splines. Ann. Stat..

[73] Phillips S.J., Anderson R.P., Schapire R.E. (2006). Maximum Entropy Modeling of Species Geographic Distributions. Ecol. Model..

[74] Hanley J.A. (1989). Receiver Operating Characteristic (ROC) Methodology: The State of the Art. Crit. Rev. Diagn. Imaging.

[75] Hart L.B., Rotstein D.S., Wells R.S., Allen J., Barleycorn A., Balmer B.C., Lane S.M., Speakman T., Zolman E.S., Stolen M. (2012). Skin Lesions on Common Bottlenose Dolphins (*Tursiops truncatus*) from Three Sites in the Northwest Atlantic, USA. PLoS ONE.

[76] Hanninger E.-M., Selling J., Heyer K., Burkhardt-Holm P. (2023). Skin Conditions, Epizoa, Ectoparasites and Emaciation in Cetaceans in the Strait of Gibraltar: An Update for the Period 2016–2020. J. Cetacean Res. Manag..

[77] Van Bressem M.-F.E.V., Flach L., Reyes J.C., Echegaray M., Santos M., Viddi F., Félix F., Lodi L., Waerebeek K.V. (2015). Epidemiological Characteristics of Skin Disorders in Cetaceans from South American Waters. Lat. Am. J. Aquat. Mamm..

[78] Haderlé R. (2022). Première Étude Des Grands Dauphins *Tursiops truncatus* Du Morphotype Dos Blanc En Guadeloupe. Master’s Thesis.

[79] Wilson B., Hammond P.S., Thompson P.M. (1999). Estimating Size and Assessing Trends in a Coastal Bottlenose Dolphin Population. Ecol. Appl..

[80] Tezanos-Pinto G., Baker C.S., Russell K., Martien K., Baird R.W., Hutt A., Stone G., Mignucci-Giannoni A., Caballero S., Endo T. (2009). A Worldwide Perspective on the Population Structure and Genetic Diversity of Bottlenose Dolphins (*Tursiops truncatus*) in New Zealand. J. Hered..

[81] Louis M., Fontaine M.C., Spitz J., Schlund E., Dabin W., Deaville R., Caurant F., Cherel Y., Guinet C., Simon-Bouhet B. (2014). Ecological Opportunities and Specializations Shaped Genetic Divergence in a Highly Mobile Marine Top Predator. Proc. R. Soc. B Biol. Sci..

[82] Hayes S., Josephson E., Maze-Foley K., Rosel P. (2017). US Atlantic and Gulf of Mexico Marine Mammal Stock Assessments-2016. NOAA Technical Memorandum NMFS-NE 238.

[83] Torres L., Rosel P., D’Agrosa C., Read A. (2003). Improving Management of Overlapping Bottlenose Dolphin Ecotypes through Spatial Analysis and Genetics. Mar. Mammal Sci..

[84] Torres L.G., Read A.J. (2009). Where to Catch a Fish? The Influence of Foraging Tactics on the Ecology of Bottlenose Dolphins (*Tursiops truncatus*) in Florida Bay, Florida. Mar. Mammal Sci..

[85] Rodriguez-Ferrer G., Appeldoorn R.S., Mignucci-Giannoni A.A., Rinaldi R., Schizas N.V. (2024). The Presence of Two Distinct Mitochondrial Lineages in the Bottlenose Dolphin (*Tursiops truncatus*) in Puerto Rico and Their Affinities with Previously Reported Lineages. Mamm. Biol..

[86] Gómez-Lobo D.A., Monteoliva A.P., Fernandez A., Arbelo M., de la Fuente J., Pérez-Gil M., Varo-Cruz N., Servidio A., Pérez-Gil E., Borrell Y.J. (2024). Mitochondrial Variation of Bottlenose Dolphins (*Tursiops truncatus*) from the Canary Islands Suggests a Key Population for Conservation with High Connectivity within the North-East Atlantic Ocean. Animals.

[87] Costa A., Fruet P., Daura-Jorge F., Simões-Lopes P., Ott P., Valiati V., Oliveira L. (2015). Bottlenose Dolphin Communities from the Southern Brazilian Coast: Do They Exchange Genes or Are They Just Neighbors?. Mar. Freshw. Res..

[88] Van Waerebeek K., Baker A.N., Félix F., Gedamke J., Iñiguez M., Sanino G.P., Secchi E., Sutaria D., van Helden A., Wang Y. (2007). Vessel Collisions with Small Cetaceans Worldwide and with Large Whales in the Southern Hemisphere, an Initial Assessment. Lat. Am. J. Aquat. Mamm..

[89] Méndez-Fernandez P., Kiszka J.J., Heithaus M.R., Beal A., Vandersarren G., Caurant F., Spitz J., Taniguchi S., Montone R.C. (2018). From Banana Fields to the Deep Blue: Assessment of Chlordecone Contamination of Oceanic Cetaceans in the Eastern Caribbean. Mar. Pollut. Bull..

[90] Fielding R., Kiszka J.J. (2021). Artisanal and Aboriginal Subsistence Whaling in Saint Vincent and the Grenadines (Eastern Caribbean): History, Catch Characteristics, and Needs for Research and Management. Front. Mar. Sci..

[91] Jung J.-L., Madon B., Boillet N., Queffelec B. (2021). Protection Des Mammifères Marins Face Aux Activités Humaines et Nouvelles Connaissances Issues Des Études de l’ADN. Actes du Colloque “Le Transport Maritime et la Protection de la Biodiversité”, Brest, 12 et 13 Décembre 2019.

[92] Sèbe M., Scemama P., Choquet A., Jung J.-L., Chircop A., Marras-Aït Razouk P., Michel S., Stiger-Pouvreau V., Recuero-Virto L. (2022). Maritime Transportation: Let’s Slow down a Bit. Sci. Total Environ..

[93] Nowacek S.M., Wells R.S., Solow A.R. (2001). Short-Term Effects of Boat Traffic on Bottlenose Dolphins, *Tursiops truncatus*, in Sarasota Bay, Florida. Mar. Mammal Sci..

[94] Janik V., Thompson P. (2006). Changes in Surfacing Patterns of Bottlenose Dolphins in Response to Boat Traffic. Mar. Mammal Sci..

[95] Hastie G.D., Wilson B., Tufft L.H., Thompson P.M. (2003). Bottlenose Dolphins Increase Breathing Synchrony in Response to Boat Traffic. Mar. Mammal Sci..

[96] Allen M.C., Read A.J., Gaudet J., Sayigh L.S. (2001). Fine-Scale Habitat Selection of Foraging Bottlenose Dolphins *Tursiops truncatus* near Clearwater, Florida. Mar. Ecol. Prog. Ser..

[97] Russell B.A., Knowlton A.R., Zoodsma B. (2001). Recommended Measures to Reduce Ship Strikes of North Atlantic Right Whales. Report Submitted to the National Marine Fisheries Service via Northeast and Southeast Implementation Teams for the Recovery of the North Atlantic Right Whale, in Partial Fulfillment of NMFS Contract 40EMF9000223.

[98] Valavi R., Guillera-Arroita G., Lahoz-Monfort J.J., Elith J. (2022). Predictive Performance of Presence-Only Species Distribution Models: A Benchmark Study with Reproducible Code. Ecol. Monogr..

