# Supplementary material for: Identification of Two Common Bottlenose Dolphin (Tursiops truncatus) Ecotypes in the Guadeloupe Archipelago, Eastern Caribbean
Source: Animals (Basel). 2025 Jan 5;15(1):108. doi: 10.3390/ani15010108 (PMC11718819; doi:10.3390/ani15010108)

Figure S1: OMMAG observation intensity map used to approximate the sampling effort associated with observation data for the two morphotypes of *T. truncatus* in the Guadeloupe archipelago. The effort values are based on the average number of monthly visits to each cell by OMMAG observers: in violet no information, in dark blue one visit, in cyan 2 to 5 visits, in green 6 to 10 visits and in yellow more than 10 visits.

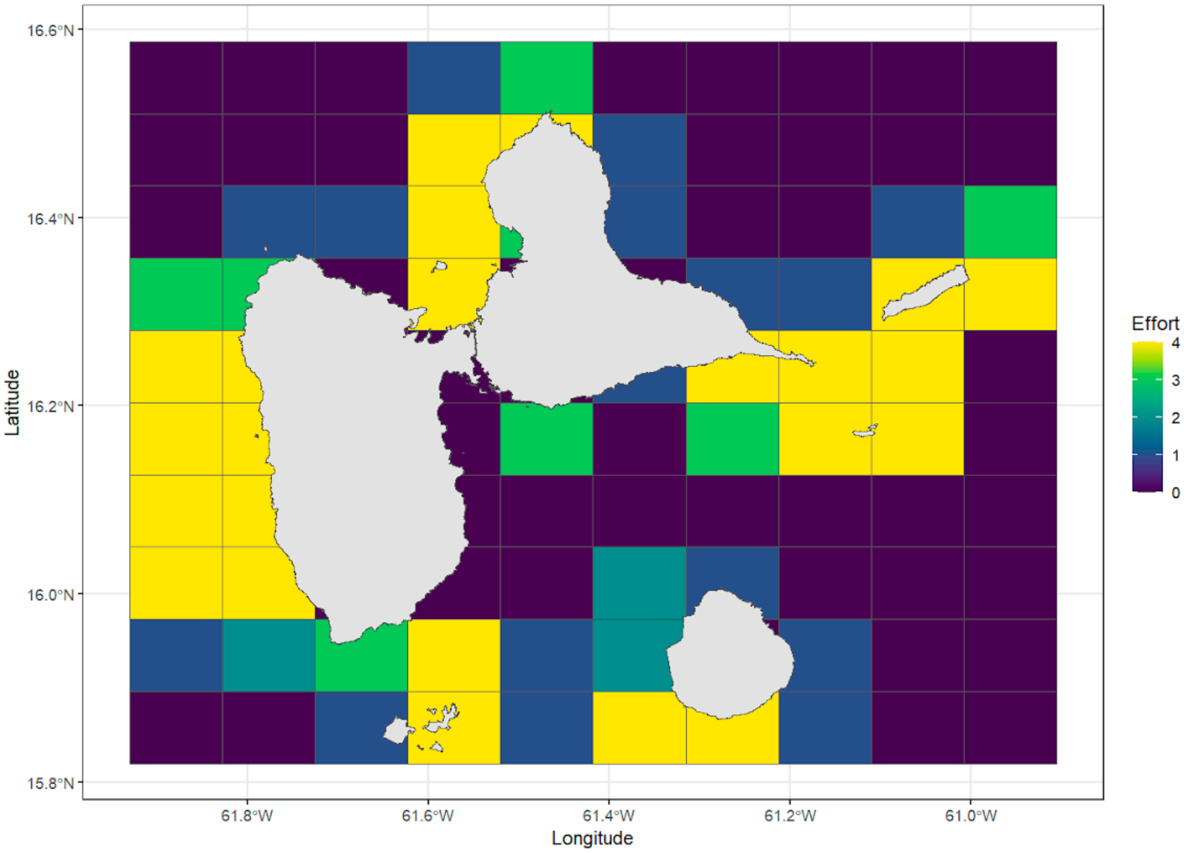

Supplement: Supplementary file 1 [file animals-15-00108-s001.zip › Figure S1.pdf]
