# Supplementary material for: Identification of Two Common Bottlenose Dolphin (Tursiops truncatus) Ecotypes in the Guadeloupe Archipelago, Eastern Caribbean
Source: Animals (Basel). 2025 Jan 5;15(1):108. doi: 10.3390/ani15010108 (PMC11718819; doi:10.3390/ani15010108)

Figure S2: Examples of depigmentation spots observed on oceanic dolphins (photographs: Laurent Bouveret, OMMAG).

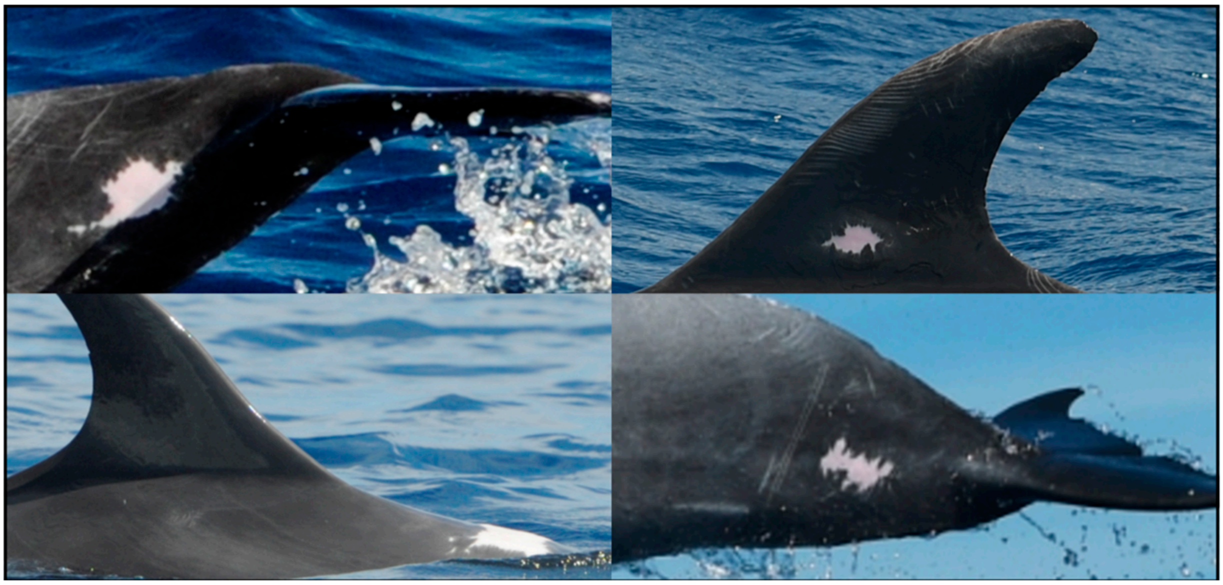

Supplement: Supplementary file 1 [file animals-15-00108-s001.zip › Figure S2.pdf]
