# Supplementary material for: Identification of Two Common Bottlenose Dolphin (Tursiops truncatus) Ecotypes in the Guadeloupe Archipelago, Eastern Caribbean
Source: Animals (Basel). 2025 Jan 5;15(1):108. doi: 10.3390/ani15010108 (PMC11718819; doi:10.3390/ani15010108)
Supplement: Supplementary file 1 [file animals-15-00108-s001.zip › Table S1.pdf]

Table S1: Summary of distinction criteria used to differentiate *T. truncatus* ecotypes in the different oceanic basins

		Distinction criteria			
**Ocean**	**References**	**Ecotypes**	**Morphology**	**Genetics**	**Ecology**
**Atlantic**	Mead & Potter, 1995; Hoelzel et al., 1998; Natoli et al., 2004; Quérouil et al. 2007; Tezanos-Pinto et al., 2009; Vollmer and Rosel, 2013; Louis et al., 2014; Costa et al., 2016; Costa et al., 2021; Simões-Lopes et al., 2019 ; Costa et al., 2022; Alexandre et al., 2024; Rodriguez-Ferrer et al., 2024	Coastal (in WNA *T. Erebennus* southern limit south-east coast of Florida*)* and oceanic<br><br>In WSA *T.t. gephyreus* (coastal)	Skull, cranial and vertebral morphologies	Separate mitochondrial lineages, with less genetic diversity in coastal populations	Distribution, diet, parasite load and group size.
**Pacific**	Hoelzel et al., 1998; Louis et al., 2014; Lowther-Thieleking et al., 2015; Costa et al., 2023	Coastal and oceanic<br>In the Northeast, *T.t. nuuanu* (oceanic)	Cranial morphology and body size	Genetic differentiation with less genetic diversity in coastal populations	Distribution, parasite load and diet
**Indian**	Hoelzel et al., 1998, Natoli et al., 2004; Särnblad et al.,2011	*T. aduncus* (coastal) & *T. truncatus* (oceanic).	Size, coloration patterns, rostrum	Genetic differentiation	Distribution and diet
