# Supplementary material for: Identification of Two Common Bottlenose Dolphin (Tursiops truncatus) Ecotypes in the Guadeloupe Archipelago, Eastern Caribbean
Source: Animals (Basel). 2025 Jan 5;15(1):108. doi: 10.3390/ani15010108 (PMC11718819; doi:10.3390/ani15010108)
Supplement: Supplementary file 1 [file animals-15-00108-s001.zip › Table S2.pdf]

Table S2: List of haplotypes used for the Caribbean dataset (accession numbers are associated)

Haplotype	Accession number	Reference study
Coastal_Bahamas_Cuba_(TtruCARA)	JN596281.1	Caballero et al. 2012
Coastal_Bahamas_Cuba_(TtruCARE)	JN596285.1	Caballero et al. 2012
Coastal_Cuba_(TtruCARL)	JN596292.1	Caballero et al. 2012
Coastal_Cuba_(TtruCARM)	JN596293.1	Caballero et al. 2012
Coastal_Cuba_(TtruCARN)	JN596294.1	Caballero et al. 2012
Coastal_Cuba_(TtruCARQ)	JN596297.1	Caballero et al. 2012
Coastal_Cuba_(TtruCARS)	JN596299.1	Caballero et al. 2012
Coastal_Cuba_Mexico_(TtruCARD)	JN596284.1	Caballero et al. 2012
Coastal_Cuba_Mexico_(TtruCARK)	JN596291.1	Caballero et al. 2012
Coastal_Cuba_Mexico_PuertoRico_VirginIslands_(TtruCARB)	JN596282.1	Caballero et al. 2012
Coastal_Mexico_(TtruCAR1TA02)	JN596320.1	Caballero et al. 2012
Coastal_Mexico_(TtruCARAA)	JN596307.1	Caballero et al. 2012
Coastal_Mexico_(TtruCARBB)	JN596308.1	Caballero et al. 2012
Coastal_Mexico_(TtruCARCC)	JN596309.1	Caballero et al. 2012
Coastal_Mexico_(TtruCARDD)	JN596310.1	Caballero et al. 2012
Coastal_Mexico_(TtruCAREE)	JN596311.1	Caballero et al. 2012
Coastal_Mexico_(TtruCARF)	JN596286.1	Caballero et al. 2012
Coastal_Mexico_(TtruCARFF)	JN596312.1	Caballero et al. 2012
Coastal_Mexico_(TtruCARU)	JN596301.1	Caballero et al. 2012
Coastal_Mexico_(TtruCARV)	JN596302.1	Caballero et al. 2012
Coastal_Mexico_(TtruCARX)	JN596304.1	Caballero et al. 2012
Coastal_Mexico_(TtruCARZ)	JN596306.1	Caballero et al. 2012
Coastal_PuertoRico_(TtruCARJJ)	JN596316.1	Caballero et al. 2012

Oceanic_Colombia_(TtruCARMM)	JN596319.1	Caballero et al. 2012
Oceanic_Colombia_Cuba_Honduras_PuertoRico_(TtruCARC)	JN596283.1	Caballero et al. 2012
Oceanic_Cuba_(TtruCARJ)	JN596290.1	Caballero et al. 2012
Oceanic_Cuba_(TtruCARO)	JN596295.1	Caballero et al. 2012
Oceanic_Cuba_(TtruCARP)	JN596296.1	Caballero et al. 2012
Oceanic_Cuba_(TtruCARR)	JN596298.1	Caballero et al. 2012
Oceanic_Honduras(TtruCARG)	JN596287.1	Caballero et al. 2012
Oceanic_Jamaica_(TtruCART)	JN596300.1	Caballero et al. 2012
Oceanic_Mexico_(TtruCARQR1)	JN596321.1	Caballero et al. 2012
Oceanic_Mexico_(TtruCARW)	JN596303.1	Caballero et al. 2012
Oceanic_Mexico_(TtruCARY)	JN596305.1	Caballero et al. 2012
Oceanic_PuertoRico_(TtruCARGG)	JN596313.1	Caballero et al. 2012
Oceanic_PuertoRico_(TtruCARH)	JN596288.1	Caballero et al. 2012
Oceanic_PuertoRico_(TtruCARHH)	JN596314.1	Caballero et al. 2012
Oceanic_PuertoRico_(TtruCARI)	JN596289.1	Caballero et al. 2012
Oceanic_PuertoRico_(TtruCARII)	JN596315.1	Caballero et al. 2012
Oceanic_PuertoRico_(TtruCARKK)	JN596317.1	Caballero et al. 2012
Oceanic_PuertoRico_(TtruCARLL)	JN596318.1	Caballero et al. 2012
Coastal_BDTPanama_(KX833116)	KX833116	Barragán-Barrera et al. 2017
Oceanic_CostaRica_(KY817220)	KY817220	Barragán-Barrera et al. 2017
Oceanic_CostaRica_(KY817221)	KY817221	Barragán-Barrera et al. 2017
