# Supplementary material for: Identification of Two Common Bottlenose Dolphin (Tursiops truncatus) Ecotypes in the Guadeloupe Archipelago, Eastern Caribbean
Source: Animals (Basel). 2025 Jan 5;15(1):108. doi: 10.3390/ani15010108 (PMC11718819; doi:10.3390/ani15010108)
Supplement: Supplementary file 1 [file animals-15-00108-s001.zip › Table S3.pdf]

Table S3: List of haplotypes used for the Atlantic dataset (accession numbers are associated)

Accession number	Reference study
JN944260	Vollmer and Rosel 2017
JN944261	Vollmer and Rosel 2017
JN944243	Vollmer and Rosel 2017
JN944244	Vollmer and Rosel 2017
JN944245	Vollmer and Rosel 2017
JN944263	Vollmer and Rosel 2017
JN944246	Vollmer and Rosel 2017
JN944262	Vollmer and Rosel 2017
JN944264	Vollmer and Rosel 2017
JN944247	Vollmer and Rosel 2017
JN944248	Vollmer and Rosel 2017
JN944249	Vollmer and Rosel 2017
JN944250	Vollmer and Rosel 2017
JN944251	Vollmer and Rosel 2017
JN944252	Vollmer and Rosel 2017
JN944259	Vollmer and Rosel 2017
JN944253	Vollmer and Rosel 2017
JN944254	Vollmer and Rosel 2017
JN944255	Vollmer and Rosel 2017
JN944257	Vollmer and Rosel 2017
GQ504051	Vollmer and Rosel 2017
AY997307	Vollmer and Rosel 2017
AY997310	Vollmer and Rosel 2017
GQ504060	Vollmer and Rosel 2017
JN944194	Vollmer and Rosel 2017
GQ504063	Vollmer and Rosel 2017
GQ504064	Vollmer and Rosel 2017
AY997311	Vollmer and Rosel 2017
JN944196	Vollmer and Rosel 2017
JN944197	Vollmer and Rosel 2017
JN944198	Vollmer and Rosel 2017
JN944199	Vollmer and Rosel 2017
JN944200	Vollmer and Rosel 2017
JN944201	Vollmer and Rosel 2017
JN944210	Vollmer and Rosel 2017
JN944211	Vollmer and Rosel 2017
JN944212	Vollmer and Rosel 2017
JN944213	Vollmer and Rosel 2017
GQ504065	Vollmer and Rosel 2017
GQ504067	Vollmer and Rosel 2017
GQ504068	Vollmer and Rosel 2017
GQ504071	Vollmer and Rosel 2017

GQ504073	Vollmer and Rosel 2017
GQ504074	Vollmer and Rosel 2017
GQ504054	Vollmer and Rosel 2017
GQ504077	Vollmer and Rosel 2017
GQ504085	Vollmer and Rosel 2017
GQ504088	Vollmer and Rosel 2017
GQ504096	Vollmer and Rosel 2017
GQ504098	Vollmer and Rosel 2017
GQ504090	Vollmer and Rosel 2017
GQ504106	Vollmer and Rosel 2017
JN944217	Vollmer and Rosel 2017
JN944219	Vollmer and Rosel 2017
JN944221	Vollmer and Rosel 2017
JN944222	Vollmer and Rosel 2017
JN944223	Vollmer and Rosel 2017
JN944224	Vollmer and Rosel 2017
JN944225	Vollmer and Rosel 2017
JN944226	Vollmer and Rosel 2017
JN944227	Vollmer and Rosel 2017
HQ383684	Vollmer and Rosel 2017
JN944230	Vollmer and Rosel 2017
JN944231	Vollmer and Rosel 2017
JN944235	Vollmer and Rosel 2017
JN944236	Vollmer and Rosel 2017
GQ504040	Vollmer and Rosel 2017
AY997308	Vollmer and Rosel 2017
GQ504049	Vollmer and Rosel 2017
AY997309	Vollmer and Rosel 2017
JN944242	Vollmer and Rosel 2017
MK105860.1	Costa et al. 2021
MK105858.1	Costa et al. 2021
MK105857.1	Costa et al. 2021
MK105876.1	Costa et al. 2021
MK105875.1	Costa et al. 2021
MK105874.1	Costa et al. 2021
MK105873.1	Costa et al. 2021
MK105872.1	Costa et al. 2021
MK105871.1	Costa et al. 2021
MK105870.1	Costa et al. 2021
MK105869.1	Costa et al. 2021
MK105868.1	Costa et al. 2021
MK105867.1	Costa et al. 2021
MK105866.1	Costa et al. 2021
MK105865.1	Costa et al. 2021
MK105864.1	Costa et al. 2021

MK105863.1	Costa et al. 2021
MK105862.1	Costa et al. 2021
MK105878.1	Costa et al. 2021
MK105877.1	Costa et al. 2021
MK105861.1	Costa et al. 2021
MK105859.1	Costa et al. 2021
MK105857.1	Costa et al. 2021
KF650783.1	Louis et al. 2014
KF650783.1	Louis et al. 2014
KF650784.1	Louis et al. 2014
KF650784.1	Louis et al. 2014
KF650785.1	Louis et al. 2014
KF650785.1	Louis et al. 2014
KF650786.1	Louis et al. 2014
KF650787.1	Louis et al. 2014
KF650787.1	Louis et al. 2014
KF650788.1	Louis et al. 2014
KF650789.1	Louis et al. 2014
KF650789.1	Louis et al. 2014
KF650790.1	Louis et al. 2014
KF650790.1	Louis et al. 2014
KF650791.1	Louis et al. 2014
KF650791.1	Louis et al. 2014
KF650792.1	Louis et al. 2014
KF650793.1	Louis et al. 2014
KF650793.1	Louis et al. 2014
KF650794,1	Louis et al. 2014
KF650795.1	Louis et al. 2014
KF650796.1	Louis et al. 2014
KF650797.1	Louis et al. 2014
KF650797.1	Louis et al. 2014
KF650798.1	Louis et al. 2014
KF650799.1	Louis et al. 2014
KF650800.1	Louis et al. 2014
KF650801.1	Louis et al. 2014
KF650802.1	Louis et al. 2014
KF650803.1	Louis et al. 2014
KF650803.1	Louis et al. 2014
KF650804.1	Louis et al. 2014
KF650805.1	Louis et al. 2014
KF650806.1	Louis et al. 2014
KF650806.1	Louis et al. 2014
KF650807.1	Louis et al. 2014
KF650808.1	Louis et al. 2014
KF650809.1	Louis et al. 2014

KF650810.1	Louis et al. 2014
KF650811.1	Louis et al. 2014
KF650812.1	Louis et al. 2014
KF650812.1	Louis et al. 2014
KF650813.1	Louis et al. 2014
KF650814.1	Louis et al. 2014
KF650815.1	Louis et al. 2014
KF650816.1	Louis et al. 2014
KF650817.1	Louis et al. 2014
KF650818.1	Louis et al. 2014
KF650819.1	Louis et al. 2014
KF650820.1	Louis et al. 2014
KF650821.1	Louis et al. 2014
KF650822.1	Louis et al. 2014
KF650823.1	Louis et al. 2014
KF650824.1	Louis et al. 2014
KF650825.1	Louis et al. 2014
KF650826.1	Louis et al. 2014
KF650827.1	Louis et al. 2014
KF650828.1	Louis et al. 2014
KF650829.1	Louis et al. 2014
KF650830.1	Louis et al. 2014
KF650831.1	Louis et al. 2014
KF650832.1	Louis et al. 2014
KF650833.1	Louis et al. 2014
KF650834.1	Louis et al. 2014
KF650835.1	Louis et al. 2014
KF650836.1	Louis et al. 2014
KF650837.1	Louis et al. 2014
DQ525388.1	Quérouil et al. 2007
DQ525387.1	Quérouil et al. 2007
DQ525386.1	Quérouil et al. 2007
DQ525385.1	Quérouil et al. 2007
DQ525384.1	Quérouil et al. 2007
DQ525383.1	Quérouil et al. 2007
DQ525382.1	Quérouil et al. 2007
DQ525381.1	Quérouil et al. 2007
DQ525380.1	Quérouil et al. 2007
DQ525379.1	Quérouil et al. 2007
DQ525378.1	Quérouil et al. 2007
DQ525377.1	Quérouil et al. 2007
DQ525376.1	Quérouil et al. 2007
DQ525375.1	Quérouil et al. 2007
DQ525374.1	Quérouil et al. 2007
DQ525373.1	Quérouil et al. 2007

DQ525372.1	Quérouil et al. 2007
DQ525371.1	Quérouil et al. 2007
DQ525370.1	Quérouil et al. 2007
DQ525369.1	Quérouil et al. 2007
DQ525368.1	Quérouil et al. 2007
DQ525367.1	Quérouil et al. 2007
DQ525366.1	Quérouil et al. 2007
DQ525365.1	Quérouil et al. 2007
DQ525364.1	Quérouil et al. 2007
FJ768019.1	Quérouil et al. 2007
FJ768018.1	Quérouil et al. 2007
FJ768017.1	Quérouil et al. 2007
FJ768016.1	Quérouil et al. 2007
FJ768015.1	Quérouil et al. 2007
FJ768014.1	Quérouil et al. 2007
FJ768013.1	Quérouil et al. 2007
FJ768012.1	Quérouil et al. 2007
FJ768011.1	Quérouil et al. 2007
FJ768010.1	Quérouil et al. 2007
FJ768009.1	Quérouil et al. 2007
FJ768008.1	Quérouil et al. 2007
FJ768007.1	Quérouil et al. 2007
FJ768006.1	Quérouil et al. 2007
FJ768005.1	Quérouil et al. 2007
FJ768004.1	Quérouil et al. 2007
FJ768003.1	Quérouil et al. 2007
FJ768001.1	Quérouil et al. 2007
DQ525362.1	Quérouil et al. 2007
DQ525361.1	Quérouil et al. 2007
DQ525360.1	Quérouil et al. 2007
DQ525359.1	Quérouil et al. 2007
DQ525358.1	Quérouil et al. 2007
DQ525357.1	Quérouil et al. 2007
DQ073717.1	Quérouil et al. 2007
DQ073716.1	Quérouil et al. 2007
DQ073715.1	Quérouil et al. 2007
DQ073714.1	Quérouil et al. 2007
DQ073713.1	Quérouil et al. 2007
DQ073712.1	Quérouil et al. 2007
DQ073711.1	Quérouil et al. 2007
DQ073710.1	Quérouil et al. 2007
DQ073709.1	Quérouil et al. 2007
DQ073708.1	Quérouil et al. 2007
DQ073707.1	Quérouil et al. 2007
DQ073706.1	Quérouil et al. 2007

DQ073705.1	Quérouil et al. 2007
DQ073704.1	Quérouil et al. 2007
DQ073703.1	Quérouil et al. 2007
DQ073702.1	Quérouil et al. 2007
DQ073701.1	Quérouil et al. 2007
DQ073700.1	Quérouil et al. 2007
DQ073699.1	Quérouil et al. 2007
DQ073698.1	Quérouil et al. 2007
DQ073697.1	Quérouil et al. 2007
DQ073696.1	Quérouil et al. 2007
DQ073695.1	Quérouil et al. 2007
DQ073694.1	Quérouil et al. 2007
DQ073693.1	Quérouil et al. 2007
DQ073692.1	Quérouil et al. 2007
DQ073691.1	Quérouil et al. 2007
DQ073690.1	Quérouil et al. 2007
DQ073689.1	Quérouil et al. 2007
DQ073688.1	Quérouil et al. 2007
DQ073687.1	Quérouil et al. 2007
DQ073686.1	Quérouil et al. 2007
DQ073685.1	Quérouil et al. 2007
DQ073684.1	Quérouil et al. 2007
DQ073683.1	Quérouil et al. 2007
DQ073682.1	Quérouil et al. 2007
DQ073681.1	Quérouil et al. 2007
DQ073680.1	Quérouil et al. 2007
DQ073679.1	Quérouil et al. 2007
DQ073678.1	Quérouil et al. 2007
DQ073677.1	Quérouil et al. 2007
DQ073676.1	Quérouil et al. 2007
DQ073675.1	Quérouil et al. 2007
DQ073674.1	Quérouil et al. 2007
DQ073673.1	Quérouil et al. 2007
DQ073672.1	Quérouil et al. 2007
DQ073671.1	Quérouil et al. 2007
DQ073670.1	Quérouil et al. 2007
DQ073669.1	Quérouil et al. 2007
DQ073668.1	Quérouil et al. 2007
DQ073667.1	Quérouil et al. 2007
DQ073666.1	Quérouil et al. 2007
DQ073665.1	Quérouil et al. 2007
DQ073664.1	Quérouil et al. 2007
DQ073663.1	Quérouil et al. 2007
DQ073662.1	Quérouil et al. 2007
DQ073661.1	Quérouil et al. 2007

DQ073660.1	Quérouil et al. 2007
DQ073659.1	Quérouil et al. 2007
DQ073658.1	Quérouil et al. 2007
DQ073657.1	Quérouil et al. 2007
DQ073656.1	Quérouil et al. 2007
DQ073655.1	Quérouil et al. 2007
DQ073654.1	Quérouil et al. 2007
DQ073653.1	Quérouil et al. 2007
DQ073652.1	Quérouil et al. 2007
DQ073651.1	Quérouil et al. 2007
DQ073650.1	Quérouil et al. 2007
DQ073649.1	Quérouil et al. 2007
DQ073648.1	Quérouil et al. 2007
DQ073647.1	Quérouil et al. 2007
DQ073646.1	Quérouil et al. 2007
DQ073645.1	Quérouil et al. 2007
DQ073644.1	Quérouil et al. 2007
DQ073643.1	Quérouil et al. 2007
DQ073642.1	Quérouil et al. 2007
DQ073641.1	Quérouil et al. 2007
