# Supplementary material for: Identification of Two Common Bottlenose Dolphin (Tursiops truncatus) Ecotypes in the Guadeloupe Archipelago, Eastern Caribbean
Source: Animals (Basel). 2025 Jan 5;15(1):108. doi: 10.3390/ani15010108 (PMC11718819; doi:10.3390/ani15010108)
Supplement: Supplementary file 1 [file animals-15-00108-s001.zip › Table S4.pdf]

Table S4: Environmental **variables used for habitat modeling and their ecological relevance**

Variable	Source	Description	Ecological Relevance
Mean bathymetry	Bio-Oracle [57]	Average water depth in the study area.	Determines habitat suitability, as bathymetry influences species distribution and prey availability.
Mean chlorophyll a concentration	Bio-Oracle [57]	Average concentration of chlorophyll a (mg/m^3), an indicator of phytoplankton abundance.	A proxy for primary productivity, influencing prey abundance for *T. truncatus*.
Minimum chlorophyll a concentration	Bio-Oracle [57]	Minimum recorded chlorophyll a concentration.	Captures seasonal or location-specific food availability.
Maximum chlorophyll a concentration	Bio-Oracle [57]	Maximum recorded chlorophyll a concentration.	Identifies areas with occasional peaks in productivity, which attract prey species.
Mean surface temperature	WordClim [56]	Average sea surface temperature (°C).	Influences species metabolism, prey distribution, and habitat preferences.
Minimum surface temperature	WordClim [56]	Minimum recorded sea surface temperature.	Highlights thermal tolerance limits and seasonality.
Maximum surface temperature	WordClim [56]	Maximum recorded sea surface temperature.	Indicates thermal extremes, which can affect habitat suitability.
Mean salinity	Bio-Oracle [57]	Average salinity levels of surface waters (psu).	Influences species distribution and habitat preferences, particularly for coastal vs. oceanic ecotypes.
Distance from the coast	Custom calculation	Euclidean distance from the nearest coastline.	Differentiates coastal and oceanic habitats.
Bottom slope	Bio-Oracle [57]	Gradient of the ocean floor, calculated from bathymetry data.	Indicates areas with steep slopes, which are often associated with upwelling and prey aggregation.
Light attenuation coefficient	Bio-Oracle [57]	Measure of light penetration through water (m^{-1}).	Affects underwater visibility and productivity, influencing species' hunting and feeding strategies.
Photosynthetically active radiation (PAR)	Bio-Oracle [57]	Light available for photosynthesis, used as a proxy for water turbidity.	Indicates water clarity, which can impact predator-prey interactions and habitat preference.
