# Supplementary material for: Identification of Two Common Bottlenose Dolphin (Tursiops truncatus) Ecotypes in the Guadeloupe Archipelago, Eastern Caribbean
Source: Animals (Basel). 2025 Jan 5;15(1):108. doi: 10.3390/ani15010108 (PMC11718819; doi:10.3390/ani15010108)
Supplement: Supplementary file 1 [file animals-15-00108-s001.zip › Table S5.pdf]

Table S5: Summary of genetic diversity indices, habitat overlaps and morphological differences between coastal and oceanic morphotypes of *T. truncatus* in the Guadeloupe archipelago.

Feature	Coastal Morphotype	Oceanic Morphotype
Genetic Diversity	Hd (Haplotype Diversity): 0.476	Hd (Haplotype Diversity): 1.000
	π (Nucleotide Diversity): 0.019	π (Nucleotide Diversity): 0.261
Population Size	Estimated at 550 individuals	Observed in 165 individuals
Haplotype Groups	Group A (in green, similar to Caribbean coastal haplotypes)	Group B (in blue, similar to oceanic haplotypes from the NEA and Azores)
Habitat Overlap	22.5% overlap in distribution patterns	77.5% exclusive to offshore areas
Habitat Suitability	Northwest and Southeast Guadeloupe	West coast and north of Guadeloupe
Exposures to Maritime Traffic	Petit Cul-de-Sac Marin and islands in transit zones	West coast exposed to international shipping
Morphological Differences	Smaller size, lighter coat, more rounded melon, more triangular dorsal fin	Larger size, darker coat, elongated falciform dorsal fin, white patches of depigmentation
